# Monitoring variability in parameter estimates for lumped parameter models of the systemic circulation using longitudinal hemodynamic measurements

**DOI:** 10.1186/s12938-023-01086-y

**Published:** 2023-04-13

**Authors:** Nikolai L. Bjørdalsbakke, Jacob Sturdy, Emma M. L. Ingeström, Leif R. Hellevik

**Affiliations:** 1 Department of Structural Engineering, Norwegian University of Science and Technology (NTNU), Richard Birkelandsvei 1a, Trondheim, Norway; 2 Department of Circulation and Medical Imaging, Norwegian University of Science and Technology (NTNU), Prinsesse Kristinas gt. 3, Trondheim, Norway

**Keywords:** Cardiovascular modeling, Model personalization, Lumped-parameter models, Parameter variability

## Abstract

**Background:**

Physics-based cardiovascular models are only recently being considered for disease diagnosis or prognosis in clinical settings. These models depend on parameters representing the physical and physiological properties of the modeled system. Personalizing these parameters may give insight into the specific state of the individual and etiology of disease. We applied a relatively fast model optimization scheme based on common local optimization methods to two model formulations of the left ventricle and systemic circulation. One closed-loop model and one open-loop model were applied. Intermittently collected hemodynamic data from an exercise motivation study were used to personalize these models for data from 25 participants. The hemodynamic data were collected for each participant at the start, middle and end of the trial. We constructed two data sets for the participants, both consisting of systolic and diastolic brachial pressure, stroke volume, and left-ventricular outflow tract velocity traces paired with either the finger arterial pressure waveform or the carotid pressure waveform.

**Results:**

We examined the feasibility of separating parameter estimates for the individual from population estimates by assessing the variability of estimates using the interquartile range. We found that the estimated parameter values were similar for the two model formulations, but that the systemic arterial compliance was significantly different ($p < 10^{-6}$) depending on choice of pressure waveform. The estimates of systemic arterial compliance were on average higher when using the finger artery pressure waveform as compared to the carotid waveform.

**Conclusions:**

We found that for the majority of participants, the variability of parameter estimates for a given participant on any measurement day was lower than the variability both across all measurement days combined for one participant, and for the population. This indicates that it is possible to identify individuals from the population, and that we can distinguish different measurement days for the individual participant by parameter values using the presented optimization method.

**Supplementary Information:**

The online version contains supplementary material available at 10.1186/s12938-023-01086-y.

## Background

Cardiovascular disease is a leading cause of loss of quality of life and premature death worldwide [1]. Although much of the pathophysiology is known, cardiovascular disease typically progresses over several years before detectable, and then often severe, symptoms emerge. Furthermore, subsequent prediction of the cardiovascular response and determining the benefits of early intervention remain challenging. Computational modeling has been proposed, by several engineers and researchers, to improve early detection and treatment of cardiovascular disease [2–4]. These models parallel similar applications in engineering. However, one of the main issues in medical applications is the estimation of model parameter values representing an individual patient (model personalization) [4].

Previous research has shown promising results for personalizing cardiovascular models [5–12] or predicting intervention effects in settings of localized cardiovascular disease and critical care [13, 14]. In this work, we investigated the application of such models as a tool for monitoring of the left ventricle and systemic circulation in apparently healthy adults at risk of developing cardiovascular disease. The parameters of hemodynamic models represent mechanical properties of the heart and blood vessels, such as contractility, compliance, and resistance, and their estimation can be seen as low-level phenotyping. Longitudinal monitoring of subtle changes in individual hemodynamics may provide means for early detection of novel risk factors and cardiovascular disease progression, which may otherwise be ignored or undetected. Furthermore, hemodynamic modeling may be used to predict changes to a given stimuli to determine the best course of treatment.

Various approaches for personalizing cardiovascular models have been demonstrated [5–12]. We focused on an approach for improving screening of apparently healthy adults at risk of cardiovascular disease in clinical practice. In this context, we identified three main factors influencing the choice of model and personalization method. First, we considered the cardiovascular physiology of interest which was defined by targeted model outputs such as central blood pressure, ventricular and aortic blood flow. Second, the objective of widespread hemodynamic monitoring limits the feasibility of acquiring detailed anatomical data on vascular networks, and thus, we considered which data may be available in a realistic clinical setting. We focused on models and personalization methods that can be accomplished from widely available non-invasive clinical measurements, such as echocardiography and continuous blood pressure monitoring. Third, we considered model performance in terms of precision, accuracy, and predictive power. A main consideration was that increased model complexity may give a better representation of underlying mechanisms, but requires more data to constrain the additional parameters [3, 15]. Another consideration, of relevance to clinical practice, was the computational complexity and time cost of evaluating more sophisticated models. Indeed, increased model complexity becomes particularly problematic in the personalization process as computational demands for estimation of personalized parameters can increase. Additionally, a more complex model with more personalizable parameters increases uncertainty in model outputs [3]. We investigated two effectively minimal models of the cardiovascular system consisting of lumped parameter representations of the left ventricle and the systemic circulation in a closed- and open-loop, respectively. In summary, the minimal approach in this work was motivated by the cardiovascular physiology of interest, the limited clinical data, and the long-term goal of enabling personalized predictions in clinical practice without a large additional burden to the patient and healthcare provider.

In this work, we developed a computationally efficient approach to personalize two minimal models based on numerical optimization to adapt the model outputs to measured data. Our approach is presented as a computationally cheaper method compared to more complex global methods. Simultaneously, we used an ensemble of estimates to account for uncertainty in the initial parameter estimates. We evaluated this modeling and personalization approach with available data from 25 individuals, with initially low physical activity levels, participating in a pilot study investigating the effects of physical activity self-monitoring on blood pressure. The participants were given advice on how much physical activity they should aim to engage in over the course of 12 weeks while monitoring their activity by wrist-worn heart rate sensors. Clinical measurements of blood pressure, volume, and flows were acquired at the beginning, middle, and end of the intervention period to detect potentially non-linear parameter changes. This study, similarly to the work of [16], investigates the change of parameters throughout an intervention period, which could give more insight into progression of disease or therapy. However, Audebert et al. focus on a parameter in response to disease progression in rats, while we monitor exercise as hypertension therapy in humans.

Our primary objective was to find personalization methods which could reliably estimate model parameter values specific for each participant and measurement day. Our evaluation of the parameter estimates used the relative variability of individual parameter estimates in comparison to the variation of parameter estimates for all participants. To this end, we express variability as the interquartile parameter range normalized by the median and refer to it as the interquartile range (IQR). Furthermore, we evaluated the consistency in parameter estimates from the closed- and open-loop models and using various pressure waveforms. The model output of primary interest was the central aortic pressure wave for monitoring of medical conditions such as hypertension. In summary, this study investigates the feasibility of using lumped parameter models with different data to detect personalized changes in model parameters after 6 to 12 weeks of exercise.

## Method

In this work, we used data on brachial arterial pressure, finger arterial pressure, pulse wave velocity (PWV), and volumetric flow in the left-ventricular outflow tract (LVOT) before and after 6 and 12 weeks of physical activity.

### Study design, setting, and participants

Personal Activity Intelligence (PAI) is a personalized and relative metric of exercise frequency, duration, and intensity based on heart rate monitoring and an accumulated score of $\ge$ 100 PAI/week is associated with higher cardiorespiratory fitness and lower cardiovascular mortality [17, 18]. We used data from a pilot randomized controlled trial to assess whether a 12-week intervention with PAI monitors increase physical activity and reduce 24-h ambulatory blood pressure in adults with elevated blood pressure [19]. A secondary aim of the trial was to collect data for computational models describing cardiovascular remodeling of physical activity. The data provided varied opportunities for personalizing models, and this work outlines our approach for model personalization. The trial was approved by Regional Committee on Medical and Health Research Ethics of Norway (Identifier: 2019/1084) and preregistered on clinicaltrials.org (Identifier: NCT 04151537).

### Data collection

All hemodynamic measurements were collected at pre- (baseline), mid- (6 weeks), and post-intervention (12 weeks), denoted 1, 2, and 3 as superscripts in the formulations, respectively.

#### Physical activity monitoring

To assess physical activity level at baseline and during the whole 12-week intervention period, all participants were provided a wrist-worn heart rate monitor without a display (LYNK2). The monitor automatically processed raw heart rate data to an aggregated weekly PAI score. The baseline period lasted one initial week directly before the intervention period. Twenty-six initially inactive participants (< 50 PAI/week based on self-reported physical activity) with elevated blood pressure (systolic $\ge$ 130 mmHg and/or diastolic $\ge$ 80 mmHg) were randomized 1:1 to an active intervention or passive control. Participants in the active intervention were provided with a mobile application for self-monitoring of PAI score and were instructed to obtain and maintain $\ge$ 100 PAI/week. Participants in the passive control were recommended to follow the World Health Organization’s physical activity guidelines of 150 min of moderate intensity or 75 min of vigorous intensity activity or any combination thereof per week [20].

#### Blood pressure recordings

Brachial and finger pressure were measured with non-invasive cuff-based devices. Brachial pressure measurement yielded momentaneous measurements of systolic and diastolic blood pressures, while the finger pressure measurements provided continuous waveforms.

Brachial pressure was recorded in two ways. First, brachial pressure was assessed in the sitting position with an automatic blood pressure monitor (TangoM2, SunTech Medical Inc) at the test station, which is from this point on referred to as office blood pressure. Second, brachial pressure was measured with a 24-h ambulatory blood pressure monitor (Oscar 2 model 250, SunTech Medical Inc), which is abbreviated to ABPM. We used the average systolic and diastolic blood pressure during waking hours in the data analyses.

Finger arterial pressure was measured in the left lateral recumbent position using Finometer PRO (FinaPres) for 4 of the participants and Non-Invasive Blood Pressure Nano (FinaPres) for the remaining participants. We synchronized all finger artery recordings with LVOT flow obtained with echocardiography.

#### Echocardiography

All participants underwent three full echocardiographic assessments (Vivid E95, VingMed). Velocity flow trace in the LVOT and stroke volume (SV) from the left ventricle were the considered most relevant measurements. Traces of LVOT flow was synchronized with finger pressure for at least three heart cycles. The first cycle was assumed to be less prone to noise and extracted as the sample cycle. We converted the velocity trace as the volumetric flow in units of milliliters per second (mL/s) to be compatible with the model formulation. Stroke volume was computed from 4D measurement of the left ventricle which was automatically segmented to determine SV and averaged over multiple heart cycles using EchoPAC (GE Healthcare). Stroke volume was used to rescale the LVOT flow cycle integral such that the cycle sample corresponded to the volume measured in 4D, which we have assumed to be a more accurate and stable measure of the SV as it was automatically averaged over multiple heart cycles.

#### Applanation tonometry

Pulse wave velocity was acquired by applanation tonometry (SphygmoCor CvMS v9, AtCor Medica). The PWV was estimated by monitoring the carotid and femoral pressure waves and computing the time of pulse propagation from the ventricle to the two points. Uncalibrated pressure cycles are reported as an image with marker points for each QRS complex. We extracted carotid pressure waveforms from the tonometry traces. The cyclic waveform data points were digitized using WebPlotDigitizer [21]. We used carotid and finger pressure waveform data for our model optimizations as continuous blood pressure data have been shown to give better estimates than using momentaneous measurements using synthetic data [22].

### Data preprocessing

The data preprocessing described in this section and the data analyses described in the subsequent sections were executed in Python (Version 3.9).

#### Finger pressure

Arterial finger pressure was synchronized to the LVOT flow signal to the precision of the closest heart beat for all three aortic flow measurement locations. The flow data were interpolated to match the time points of the pressure recording to ensure the same frequency and enable applying a standard numeric solver to the paired data. The pressure cycle was rescaled to match ambulatory blood pressure to use the finger pressure waveform as a proxy of more central waveforms.

#### Tonometry traces

The tonometry traces were of varying quality and we assessed all cycles manually before determining which to include in the analyses. The waveforms were assessed visually and discarded if they were obviously distorted, lacked any signs of the dicrotic notch plateau, or did not represent complete heart cycles. The remaining cycles, each representing a heartbeat, were normalized to a uniform scale, averaged, and subsequently scaled to match ambulatory blood pressure. The cycle lengths were averaged to estimate the heart rate, and the final pressure cycle was standardized to this heart rate.

### Pressure and flow waveform alignment

None of the collected pressure and flow waveforms were collected at the same sampling rate which was required by the chosen estimation algorithm. Matching time points ensures equal weighting of the two waveform signals due to number of contributing residuals in an optimization context. When pairing flow and the finger pressure signals, the heart cycle was determined by identifying the distance between start of upstroke for the first flow cycle and to the next start of upstroke. These time points were then used to extract a corresponding pressure cycle from the continuous pressure recording. The flow signal was linearly interpolated to match the time points of the finger pressure. For the carotid waveforms, the data with the largest mean interpoint distance in time had their measurement points rearranged to be evenly distributed over the corresponding heart cycle. Afterwards, both pressure and flow signals were interpolated to the new uniformly distanced time points. The number of total data points for the flow and carotid pressure waveforms were often comparable and therefore the number of total discretized points were not changed considerably in most cases due to interpolation. All pressure measurements were linearly rescaled so that the maximum and minimum of the waveform matched the ambulatory blood pressure systolic and diastolic values. However, in a single set of measurements the ambulatory blood pressure was missing and office blood pressure was used instead.

In both cases, the flow cycle length in time was rescaled to have the same heart rate as the pressure sample, and to have the same SV as recorded in 4D echocardiography mode. In cases where 4D SV were missing, we used the SV calculated by EchoPac from the LVOT flow. All pressure and flow cycles were aligned to start at systolic upstroke.

### Models

In this work, we applied two models of the left ventricle and systemic circulation. One closed-loop model in which the venous pressure and volume was estimated as a model prediction and one open-loop model which assumed fixed venous pressure and left atrial pressure. The parameters chosen for personalization in our models are shown in Table 1.

#### Closed-loop model

**Fig. 1:**
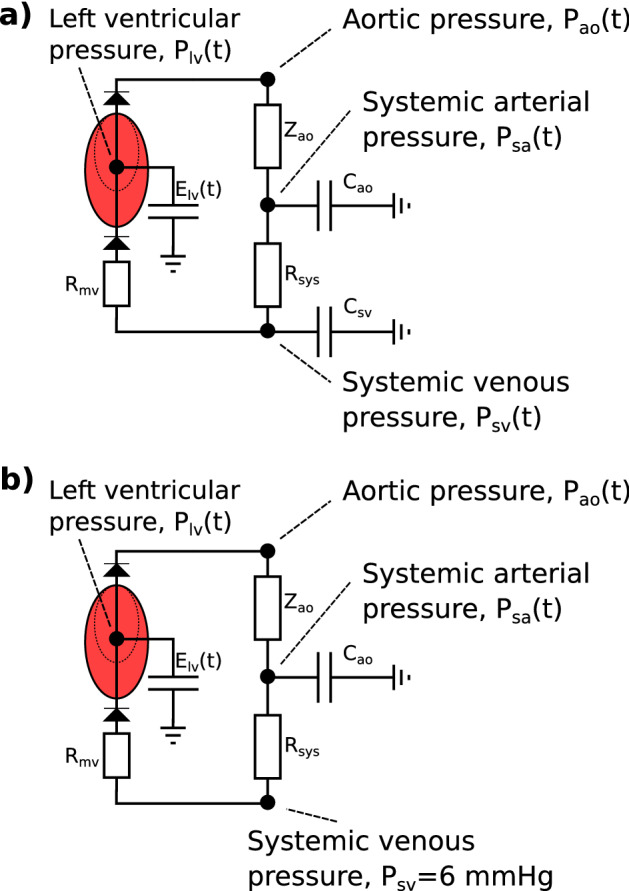
**a** The closed-loop, lumped parameter model of the left ventricle, systemic arteries, and veins. **b** The open-loop lumped parameter model of the left ventricle and systemic arteries. The circuit equivalent formulation of the models are depicted with the pressures and most of the mechanical parameters used to describe the systemic circulation. The venous compartment is volumeless and only partially described in the open-loop model. Adapted from Bjørdalsbakke et al. [22], and used under CC-BY 4.0

The simplified closed-loop model used for personalization has been presented by Bjørdalsbakke et al. [22], and is based on similar models by Segers, Smith and Stergiopulos et al. [2, 23–26]. An illustration of our version of the model is provided in Fig. 1.

The closed-loop model is described by a system of differential equations characterizing the behavior of the stressed volumes of the ventricle, arteries, and veins. All flows were computed as the pressure gradient across resistances between the model compartments. Each separate compartment was modeled using a linear relationship between pressure and volume. See “Model equations” section for the full mathematical model description.

**Table 1:** The closed-loop model parameters are listed with their corresponding symbols and units. The same parameters are used to describe the open-loop model except for $C_{sv}$ and $V_{tot}$

Symbol	Description	Unit
$C_{ao}$	Systemic arterial compliance	$\mathrm {\frac{mL}{mmHg}}$
$C_{sv}$	Systemic venous compliance	$\mathrm {\frac{mL}{mmHg}}$
$E_{max}$	Maximal left ventricular elastance	$\mathrm {\frac{mmHg}{mL}}$
$E_{min}$	Minimal left ventricular elastance	$\mathrm {\frac{mmHg}{mL}}$
$R_{mv}$	Mitral valve resistance	$\mathrm {\frac{mmHg \ s}{mL}}$
$R_{sys}$	Total peripheral resistance	$\mathrm {\frac{mmHg \ s}{mL}}$
*T*	Heart period	s
$t_{peak}$	Time of peak ventricular elastance	s
$V_{tot}$	Total stressed blood volume	mL
$Z_{ao}$	Characteristic impedance of the aorta	$\mathrm {\frac{mmHg \ s}{mL}}$

#### Open-loop model

The open-loop model formulation is identical to the closed-loop model, except that the venous compartment is removed. This means the venous compliance parameter is replaced by a fixed venous pressure value, which in turn means that the total stressed blood volume parameter fluctuates. The model is identical to the one used by Stergiopulos et al. [24], but we set the constant venous pressure to $P_{sv} = 6.0$ mmHg, which is a value within the range of a normal central venous pressure (CVP) [27, 28]. See Fig. 1 for an illustration of the model. Additional details of the mathematical description can be found in “Model equations” section.

#### Model output

The model outputs such as pressure and flow are denoted as $y(t, \varvec{\theta })$ to emphasize that each output varies with time *t* and the parameter vector $\varvec{\theta }$. From these model predictions, estimates of clinical measurements can be derived. The model predictions were computed numerically using SciPy’s implementation of the 4th-order Runge–Kutta (RK4) method to integrate the differential equations [29]. The model was solved for 10 heart cycles, and the tenth cycle was taken as the model prediction.

### Parameter estimation

The inference method for the parameters was based on a local optimization method, similar to the work outlined in [22]. We preferred a local optimization method as it is most feasible to apply to routine screening in clinical practice as global methods often demand higher computational costs. The SciPy implementation of the Trust-Region Reflective Algorithm (TRRA) was chosen as it supports the use of bounds for different parameters [29], which can constrain the parameter space to more physiologically realistic parameters.

Both models take a vector of parameters $\varvec{\theta }$ and generate outputs $y(t_k,\varvec{\theta })$ at time point *k*. A real measurement at the same time point can be described as being composed by this model output and other terms of the form

1 $$\begin{aligned} y^m_k = y(t_k, \varvec{\theta }) + E_k, \end{aligned}$$

where $E_k$ is a measurement error or noise. However, to infer the parameters which reproduce a measured set of data, a problem on the form

2 $$\begin{aligned} \hat{\varvec{\theta }} = \mathrm {arg \ min} \ J(\varvec{\theta }) \end{aligned}$$

must be solved. Here, *J* is a cost function which characterizes the optimization problem. In this work, we focused on non-linear least squares optimization.

The TRRA is dependent upon a set of initial parameter guesses $\varvec{\theta }$ where the *i*th component of the vector is $\theta _{i}$. We found personalized parameter estimation by applying the TRRA in a five-step procedure. In short, the procedure can be described as follows:

1. Use the TRRA to make 30 parameter estimates from 30 different sets of initial guesses.
2. Take the initial parameter guess which yields the lowest cost function value estimate and create a new uniform distribution centered on these parameter values.
3. Make 20 new initial guesses based on the best previous guess and produce 20 new sets of estimates, we call this set of parameters $\varvec{\Theta }_{\text{step2}}$.
4. Make a selection based on the best cost function values from Step 3, $\varvec{\Theta }^{p,d,k}_{\text{filtered}}$, where *k* denotes the *k*th filtered estimate, and *p* refers to participant, while *d* represents pre-, mid-, and post-intervention measurement days encoded as 1, 2, and 3.
5. Compute the final parameter estimate as the mean of the estimates from Step 3, such that $\hat{\theta }^{p,d}_{\text{mean},i} = \frac{1}{N_k} \sum _k \Theta ^{p,d,k}_{\text{filtered},i}$. $N_k$ is the number of remaining estimated parameter vectors in $\varvec{\Theta }^{p,d}_{\text{filtered}}$ after filtering.

To find the best parameter estimates, guesses for each component of the parameter vector were sampled from a uniform distribution with a lower bound $\theta _{\text{lower},i}$ and upper bound $\theta _{\text{upper},i}$. Consequently, we sampled the initial parameter guesses in Step 1 as

3 $$\begin{aligned} \theta _{i} = U(\theta _{\text{lower},i}, \theta _{\text{upper},i}), \end{aligned}$$

where *U*(*a*, *b*) is a continuous uniform distribution, with upper and lower bound *a* and *b*. Thirty sets of initial parameters sets were sampled and used to optimize the cost function $J(\varvec{\theta })$. In Step 2, the initial parameter guess resulting in the optimization with the smallest cost function value was taken as a new reference set $\varvec{\theta }_{\text{min}}$ to sample 20 more guesses, in Step 3, from a uniform distribution with limits 10% below and above the components of the reference parameter set as in

4 $$\begin{aligned} \theta _{i} = U(0.9 \theta _{\text{min},i}, 1.1 \theta _{\text{min},i}). \end{aligned}$$

This procedure resulted in multiple sets of estimated parameters $\hat{\varvec{\theta }}$ which in Step 4 were filtered by only accepting the results with a cost function value smaller than or equal to the mean value of the 20 optimized parameter sets from Step 3. Lastly, in Step 5, the mean of the selected parameter sets were taken as the final parameter estimate for participant *p* on measurement day *d*, $\hat{\varvec{\theta }}^{p,d}_{\text{mean}}$.

The cost function was designed to adapt the model to the pressure and flow waveforms, SV, and ambulatory blood pressure. If we let $P^m_{k}$ denote the measured pressure and $P_\mathrm{{ao},k}$ denotes the model output aortic pressure at time point *k*, while $Q^m_k$ and $Q_k$ denote the aortic flow, then the cost function can be expressed as

5 $${\begin{matrix} J(\varvec{\theta }) & = \sum\limits_{i}^{N} \left( \frac{P^m_i - P_{\text{ao},i}}{K_\text{p}} \right) ^2 + \sum _i^N \left( \frac{Q^m_{\text{ao},i} - Q_{\text{ao},i}}{K_\text{q}} \right) ^2 \\ & \quad+ \frac{7.5^2 N^2}{40^2} \left[ \left( \frac{P^m_{\text{sys}} - P_{\text{sys}}}{K_{\text{p,sys}}} \right) ^2 + \left( \frac{P^m_{\text{dia}} - P_{\text{dia}}}{K_{\text{p,dia}}} \right) ^2 \right] \\ & \quad+ \frac{7.5^2 N^2}{40^2} \left[ \left( \frac{\text{SV}^m-\text{SV}}{K_{\text{SV}}} \right) ^2 + \frac{1}{9} \left( \frac{6.0 - \text{MVP}}{K_{\text{MVP}}} \right) ^2 \right] . \end{matrix}}$$

Here, *N* is the number of time points in the waveform sample, $P_{sys}$ and $P_{dia}$ are the systolic and diastolic values of the pressure waveform, $\text{SV}^m$ is the SV corresponding to the area under the flow waveform, and the *m* superscript denotes a real measurement. $\text{SV}$ as determined by the model is the SV calculated as the maximal change of volume in the left ventricle throughout the final heart cycle. The final term constrained the mean venous pressure ($\text{MVP}$) computed by the model to approximately 6 mmHg, which is a value within the range of a normal central venous pressure (CVP) [27, 28]. The model only models the $P_{sv}$ which is the averaged pressure of all veins and is not actually tied to a vein in particular. We calculate the mean value of the pulsatile $P_{\text{sv}}$ signal to create the mean venous pressure indice $\text{MVP}$. We thereafter approximate the $\text{MVP}$ indice to be equal to the CVP as this influences ventricular filling, and the is the model concept most closely related to CVP. All constants *K* are scaling constants with a magnitude similar to a reference level for the different measurement types. For further details, settings, and bounds for the optimization routine, see Optimization algorithm details. The cost function was identical for both models, except for the final venous pressure term, which was not applicable to the open-loop model. The weights $\frac{7.5^2 N^2}{40^2}$ and $\frac{2.5^2 N^2}{40^2}$ were manually tuned to assign more weight to the pressure extrema values and clinical indices as these are particularly important in clinical settings, and perhaps also more likely to express cardiovascular remodeling. The number 40 was chosen as a reference scale since the number of data points in the carotid waveform and LVOT flow signals were usually close to this number before interpolation.

Previous optimization of parameters for the closed-loop model using aortic pressure and flow shows that $E_{\text{min}}, R_{\text{mv}}$ and $Z_{\text{ao}}$ are among the most challenging parameters to estimate in synthetic data produced by the model itself with Gaussian noise [22]. These three parameters were found to be least sensitive to aortic pressure when applying a sensitivity analysis to the model. In the parameter optimization routine used, these parameters were therefore not prioritized for attempted personalization. The remaining parameters in Table 1 were chosen for estimation, except for *T* which was estimated directly from the waveform cycle lengths. Despite the low sensitivity value, $Z_{\text{ao}}$ was included for optimization since initial attempts at optimizing the model to real data indicated that this improved the model’s ability to recreate the pressure waveform during systole. For the open-loop model, the same parameters were personalized as for the closed-loop model, except for $V_{\text{tot}}$ and $C_{\text{sv}}$. The mitral valve resistance was fixed to be $R_{mv} = 0.02 \frac{\mathrm {mmHg \ s}}{\text{mL}}$. $E_{\min }$ was fixed to 0.055 or 0.06 depending on whether or not the systolic pressure was below 140 mmHg, see Optimization algorithm details for further details.

All model output waveforms incorporated in the cost function were aligned with pressure and flow data by enforcing that the model outputs always started at beginning of systole. No single parameter determines the start of systole in the model, so this was done by translating the waveforms in time until they started at the correct value.

### Post-processing of parameter estimates

All computed parameters were normalized by body surface area (BSA) computed as

6 $$\begin{aligned} \text{BSA} = \sqrt{\frac{ \text{Height} \cdot \text{Weight}}{3600}}. \end{aligned}$$

All participants had height and weight measured at pre-intervention, while weight was also measured at mid- and post-intervention and BSA updated accordingly. All parameter estimates, except for $t_{peak}$, have been BSA indexed as a normalization to account for variations in body size for parameters, which may be assumed to be body size dependent.

Due to noisy or missing data or missing synchronization data, we could not compute parameters for all study participants. Thus, we defined two data sets, both included the LVOT flow trace converted to volumetric flow, the ambulatory blood pressure values and stroke volume. The first data set included the finger pressure waveform scaled by ambulatory blood pressure and synchronized with the LVOT flow cycle, while the second data set included the averaged carotid pressure waveform scaled by ambulatory blood pressure paired with the LVOT flow cycle.

The data sets based on finger pressure had 50 eligible measurements across all participants and measurement days, while the carotid pressure had eligible 62 measurements. For finger pressure, 9 participants could be identified at all three measurement days, whereas 14 participants were identified for carotid pressure. Seven of these participants had eligible measurements for both pressures. The total number of unique study participants in the data set was 25 when counting all data sets where all three measurement days were not present. For all eligible participants, and for both models, we estimated $E_{\text{max}}$, $R_{\text{sys}}$, $C_{\text{ao}}$, $t_{\text{peak}}$, $Z_{\text{ao}}$. For the closed-loop model, $C_{\text{sv}}$ and $V_{\text{tot}}$ were also estimated.

The variability within estimates for each individual participant and the population as a whole were assessed by computing the interquartile range and dividing by the median value. In this manuscript, we refer to this as the interquartile ratio (IQR):

7 $$\begin{aligned} \text{IQR} = \frac{\theta _{i,75\%} - \theta _{i,25\%}}{\hat{\theta }_{i,\text{median}}}, \end{aligned}$$

where the 25% and 75% subscripts refer to the corresponding percentiles. The means for the parameters over all participants and all measurement days were computed to also assess if there were differences in estimates between model formulations and pressure waveforms.

We used IQR to assess estimate variability in three different contexts. First, we investigated the variability in estimates for single participants on any measurement day, for which we calculated the IQR based on the set of estimates $\varvec{\Theta }_{\text{filtered}}$ yielded from a single set of raw data, i.e., a given participant on a given measurement day. The median IQR for these estimates was found, and the first and third quartiles were interpreted as a measure of variability in the IQR for each participant on any measurement day. We computed the IQR for all eligible participants whether they had 1, 2 or 3 available measurement days. We refer to this as the “Single day IQR”.

Second, the variation across an individual over all measurement days *d* was assessed for each individual *p*. The three estimated sets $\varvec{\Theta }_{\text{filtered}}$ resulting in parameter estimates $\hat{\varvec{\theta }}^{p,1}_{\text{mean}}$, $\hat{\varvec{\theta }}^{p,2}_{\text{mean}}$, and $\hat{\varvec{\theta }}^{p,3}_{\text{mean}}$ for each participant *p* were combined to make one common set of parameters $\varvec{\Theta }^{p}_{\mathrm {all \ days}}$ containing the best optimized parameter estimates across all measurement days for each individual. Only participants whose parameters could be estimated for all 3 days were included. For each participant, the IQR was computed based on the set $\varvec{\Theta }^{p}_{\mathrm {all \ days}}$, and afterwards the median IQR and first and third quartiles were determined. We named this quantity the “Multiple day IQR”.

Third, we computed the final parameter estimates $\hat{\varvec{\theta }}^{p,d}_{\text{mean}}$ for all participants on all measurements days and subsequently collected these in a set for which we computed the IQR value. This IQR value was interpreted as the variability in parameters across the population and across all measurement days. All participants were included, and this IQR measure is referred to as the “Population IQR”. This quantity is not presented with standard deviations as it is a single value characterizing the study population. All three IQR analyses were repeated for both models and the data sets with pressure waveform measured in either the carotid artery or finger artery.

### Other methods for estimating model parameters

The arterial parameter estimates from the personalization could be compared to estimates made with more traditional methods to estimate these. Arterial compliance can be estimated by

8 $$\begin{aligned} \tilde{C}_{\text{ao}} \approx \frac{\mathrm {SV^m}}{\mathrm {PP^m}}, \end{aligned}$$

where $\text{PP}$ is the brachial artery pulse pressure as a proxy for more central pressure. Similarly, the vascular resistance can be estimated by

9 $$\begin{aligned} \tilde{R}_{sys} \approx \frac{\mathrm {MAP^m}}{\mathrm {CO^m}}, \end{aligned}$$

where $\text{CO}$ is cardiac output and $\text{MAP}$ is mean arterial pressure calculated as the mean of the pressure waveform scaled with brachial pressure.

### Quality of waveform optimizations

To assess whether the estimated model parameters could recreate the waveforms and, especially, the other indices included in the cost function accurately, we examined the unscaled residuals between model predictions and data. Instead of listing each residual for every participant and measurement day, we assessed the mean absolute value of residuals on each measurement day for both data sets and model formulations separately. The model outputs used to compute the residuals are the outputs based on the final parameter set based on the averaged parameters from the best optimization sets, $\hat{\theta }^{p,d}_{\text{mean},i}$.

### Summary

From a pilot randomized controlled trial on self-monitoring of physical activity, blood pressure and echocardiography data for initially inactive adults were available. We implemented a closed- and open-loop model of the left ventricle and systemic circulation in Python, and optimized these using local methods to paired data of pressure and flow waveforms, including $\text{SV}$. Pressure waveforms collected by non-invasive finger pressure measurement and carotid applanation tonometry were paired with aortic flow data and applied to parameter estimations for the trial participants. We computed the variability in estimates for each individual and the population as whole using our definition of the IQR and assessed how well the parameters of participants and possible parameter changes could be resolved from the population.

## Results

The results in this section are based on analysis of parameter estimates for individuals over multiple measurement days (model personalization). We computed estimates for both a closed- and an open-loop model, but added an additional dimension in doing so for two different data sets including either the carotid or finger pressure waveforms.

### Parameter estimate variability in individuals compared to the population

**Fig. 2:**
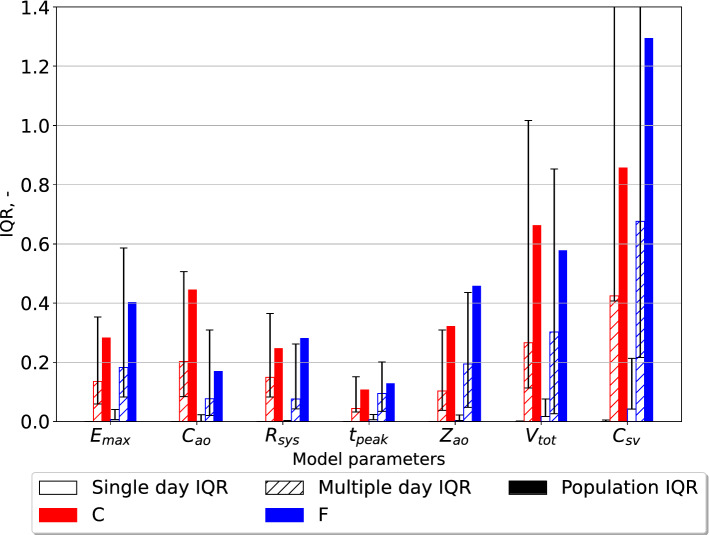
The interquartile ratio (IQR) computed for the parameters of the closed-loop model. The “Single day IQR” which is based on the median IQR values for the estimates for single individuals on any measurement day are included. The whiskers indicate first and third quartiles. The variation in individuals over all measurement days are displayed as “Multiple day IQR”. The final parameter estimates for all individuals in the different data sets yield the “Population IQR”. “F” indicates the data set with finger pressure waveform, and “C” indicates the data set with the carotid pressure waveform. The IQR refers to the difference between the upper and lower quartiles divided by the mean parameter value. $E_{\text{max}}$ is the maximal left-ventricular elastance, $C_{\text{ao}}$ is the systemic arterial compliance, $R_{\text{sys}}$ is the total peripheral resistance, $t_{\text{peak}}$ is the time of maximal ventricular elastance, $Z_{\text{ao}}$ is the characteristic aortic impedance, $V_{\text{tot}}$ is the total stressed blood volume, and $C_{\text{sv}}$ is the systemic venous compliance

**Fig. 3:**
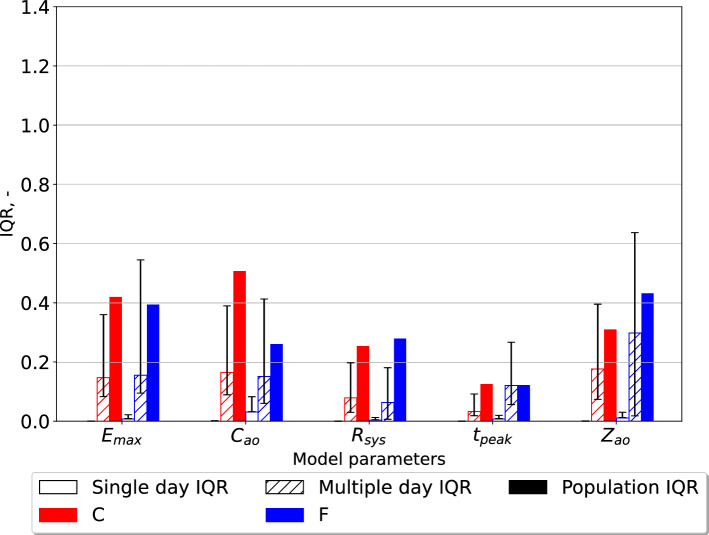
The interquartile ratio (IQR) computed for the parameters of the open-loop model. The “Single day IQR” which is based on the median IQR values for the estimates for single individuals on any measurement day are included. The whiskers indicate first and third quartiles. The variation in individual participants over all measurement days are displayed as “Multiple day IQR”. Final parameter estimates for all individuals in the different data sets yield the “Population IQR”. “F” indicates the data set with finger pressure waveform, and “C” indicates the data set with the carotid pressure waveform. The IQR refers to the difference between the upper and lower quartiles divided by the mean parameter value. $E_{\text{max}}$ is the maximal left-ventricular elastance, $C_{\text{ao}}$ is the systemic arterial compliance, $R_{\text{sys}}$ is the total peripheral resistance, $t_{\text{peak}}$ is the time of maximal ventricular elastance, and $Z_{\text{ao}}$ is the characteristic aortic impedance

**Fig. 4:**
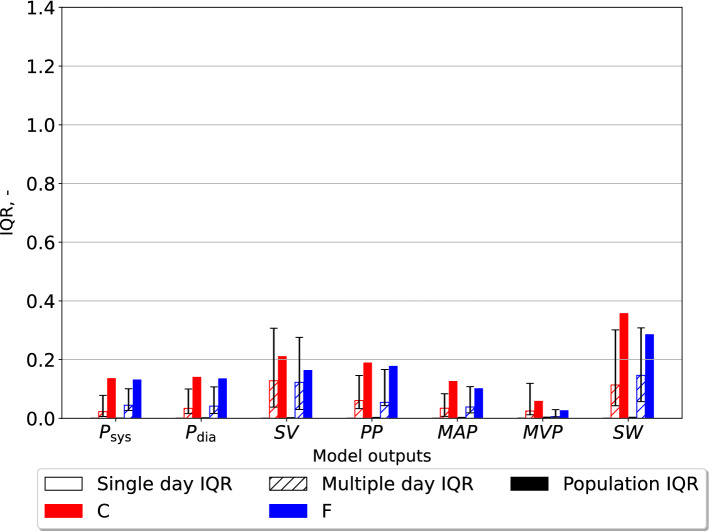
The interquartile ratio (IQR) computed for model outputs generated by the closed-loop models, each model instance optimized for one individual. The “Single day IQR” is based on the median IQR values for the model outputs from the variation in parameter estimates for single individuals on any measurement day. The whiskers indicate first and third quartiles. The variation in the best optimized sets of model outputs from individuals over all measurement days are displayed as “Multiple day IQR”. The model outputs based on the final parameter estimates for all individuals in the different data sets yield the “Population IQR”. “F” indicates the data set with finger pressure waveform, and “C” indicates the data set with the carotid pressure waveform. The IQR refers to the difference between the upper and lower quartiles divided by the mean parameter value. $P_{\text{sys}}$ is the systolic aortic pressure, $P_{\text{dia}}$ is the diastolic aortic pressure, $\text{SV}$ is the stroke volume indexed by body surface, $\text{PP}$ is the aortic pulse pressure, $\text{MAP}$ is the mean arterial pressure, $\text{MVP}$ is the mean venous pressure, and $\text{SW}$ is the left-ventricular stroke work

**Fig. 5:**
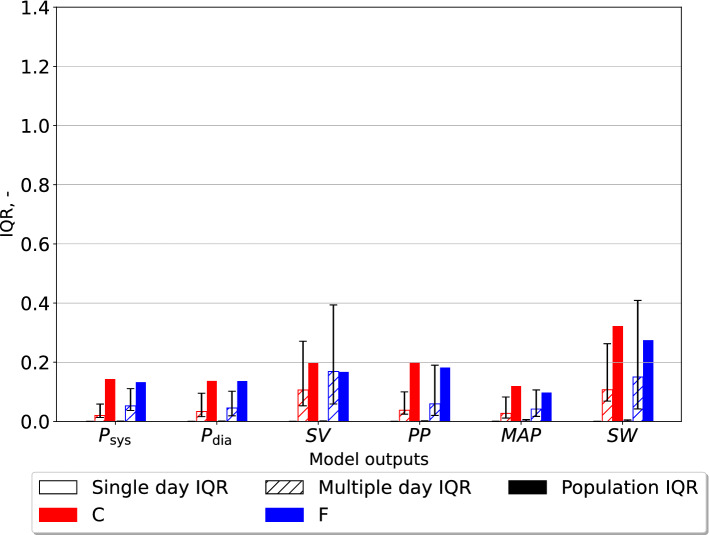
The interquartile ratio (IQR) computed for model outputs generated by the open-loop models, each model instance optimized for one individual. The “Single day IQR” is based on the median IQR values for the model outputs from the variation in parameter estimates for single individuals on any measurement day. The whiskers indicate upper and lower quartiles. The variation in the best optimized sets of model outputs from individuals over all measurement days are displayed as “Multiple day IQR”. The model outputs based on the final parameter estimates for all individuals in the different data sets are used to compute bars labeled as the “Population IQR”. “F” indicates the data set with finger pressure waveform, and “C” indicates the data set with the carotid pressure waveform. The IQR refers to the difference between the upper and lower quartiles divided by the mean parameter value. $P_{\text{sys}}$ is the systolic aortic pressure, $P_{\text{dia}}$ is the diastolic aortic pressure, $\text{SV}$ is the stroke volume indexed by body surface, $\text{PP}$ is the aortic pulse pressure, $\text{MAP}$ is the mean arterial pressure, and $\text{SW}$ is the left-ventricular stroke work

The $\text{IQR}$ as defined by equation (7) was calculated for the variation in estimates for the population given either the finger pressure or carotid pressure waveform. Similarly, the IQR scores for all individuals across all measurement days were expressed by the median IQR and variation presented as the first and third quartiles. The IQR values were calculated for both the closed- and open-loop models and the results are shown in Figs. 2 and 3, respectively. Figures 2–5 display the IQR for several model parameters and outputs. The IQR is unitless, but otherwise the units used are the same as in Table 1 for model parameters, only BSA indexed, and hence divided by $m^2$ unless otherwise noted. Only $t_{\text{peak}}$ is not BSA indexed.

The IQR was computed for different model outputs which are shown in Figs. 4, 5, for the closed- and open-loop models, respectively. All pressures $P_{\text{sys}}$, $P_{\text{dia}}$, $\text{PP}$ (pulse pressure), $\text{MAP}$ (mean arterial pressure) and $\text{MVP}$ (mean venous pressure) are measured in units of mmHg. Stroke volume *SV* is given in mL, while stroke work *SW* is expressed in mmHg$\cdot$mL.

**Fig. 6:**
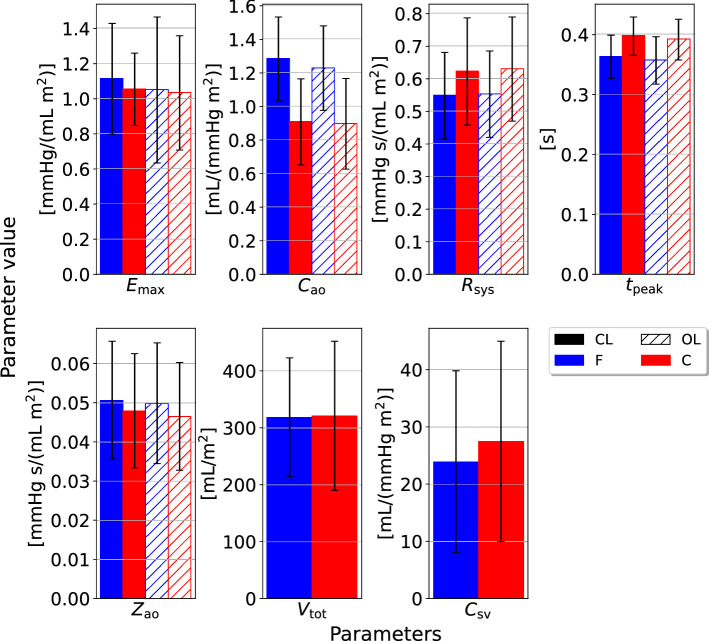
The mean parameter estimates, and standard deviations, for all individuals in the different data sets. These results originate from estimates made for the closed-loop (“CL”) and open-loop (“OL”) models, respectively. “F” indicates the finger pressure waveform, and “C” indicates the data set with the carotid pressure waveform. The y-axis represents model parameters and units listed in Table 1 as BSA indexed values, with the exception of $t_{\text{peak}}$ which is given in units seconds, s. $E_{\text{max}}$ is the maximal left-ventricular elastance, $C_{\text{ao}}$ is the systemic arterial compliance, $R_{\text{sys}}$ is the total peripheral resistance, $t_{\text{peak}}$ is the time of maximal ventricular elastance, $Z_{\text{ao}}$ is the characteristic aortic impedance, $V_{\text{tot}}$ is the total stressed blood volume, and $C_{\text{sv}}$ is the systemic venous compliance

**Fig. 7:**
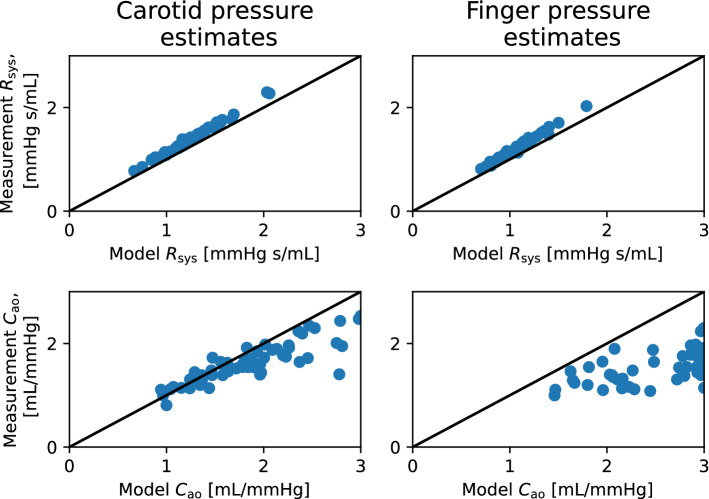
The scatter plots of closed-loop model parameter estimates for total peripheral resistance $R_{\text{sys}}$ (top panels) and arterial compliance $C_{\text{ao}}$ (bottom panels), compared to estimates from conventional methods, using the data sets with the carotid (left panels) and the finger pressure (right panels) waveforms. Model estimates are on the x-axes, while conventional estimates are on the y-axes

### Parameter estimates compared between different data sources

The mean parameter values scaled by BSA were compared for the different pressure waveforms (finger and carotid) and for the closed- and open-loop models. The results are displayed as mean values with standard deviations in Fig. 6. There are minor differences between the estimate averages, especially between model formulations. The open-loop models have on average a marginally lower $E_{\text{max}}$ than the closed-loop model. The estimate differences are larger between the different waveforms, where for example the $C_{\text{ao}}$ estimates are on average higher for the finger pressure waveform than the carotid pressure waveform.

Figure 6 contains the results from the complete case analysis of both models and pressure waveforms. We also picked the 47 paired samples where both pressure waveforms were available for the same participant and compared the means in Table 2. The function ttest() from the Pingouin Python library version 0.5.1 was used to perform a paired sample t-test and compute the mean difference and 95% confidence interval between finger and carotid pressure [30]. The difference between sample means was found to be statistically significant for both models, see Table 2. The paired sample differences for arterial compliance $C_{\text{ao}}$ were computed and the 5th, 25th, 50th, 75th and 95th percentiles were presented to give an indication of the distribution of differences not being extremely asymmetrical. For the closed-loop model these percentiles were $-$0.005, 0.233, 0.430, 0.575, and 0.771, respectively, while for the open-loop model they were 0.035, 0.186, 0.380, 0.538, and 0.709 in units mL / (mmHg$\cdot \text{m}^2$). The paired samples mean $C_{\text{ao}}$ estimates were 1.275 and 0.876 for the closed-loop model with the finger pressure waveforms and carotid pressure waveforms, respectively. Similarly, for the open-loop model the finger pressure-based sample mean was 1.221, while the carotid pressure-based sample mean was 0.860. Paired t-tests for other parameters can be seen in the supplementary materials.

**Table 2:** Mean arterial compliance $C_{\text{ao}}$ parameter values averaged over different samples of measurements for both model formulations and choice of data set. The *p*-value and 95% confidence interval (CI 95%) are obtained by a two-tailed t-test for paired data comparing the mean parameter values using the finger and carotid pressure waveforms, respectively, within the same model

Model	Samples	Mean $C_{\text{ao}}$ w/	Mean $C_{\text{ao}}$ w/	95% CI for	*p*-value
		finger pressure	carotid pressure	mean difference	
		[mL/(mmHg$\cdot \text{m}^2$)]	[mL/(mmHg$\cdot \text{m}^2$)]	[mL/(mmHg$\cdot \text{m}^2$)]	
Closed-loop	Mixed	1.28	0.91	–	–
Closed-loop	Paired	1.28	0.88	[0.33, 0.47]	$p<1.0e-14$
Open-loop	Mixed	1.23	0.90	–	–
Open-loop	Paired	1.22	0.86	[0.29, 0.43]	$p<1.0e-13$

### Comparison of arterial parameter estimates to other estimation methods

**Fig. 8:**
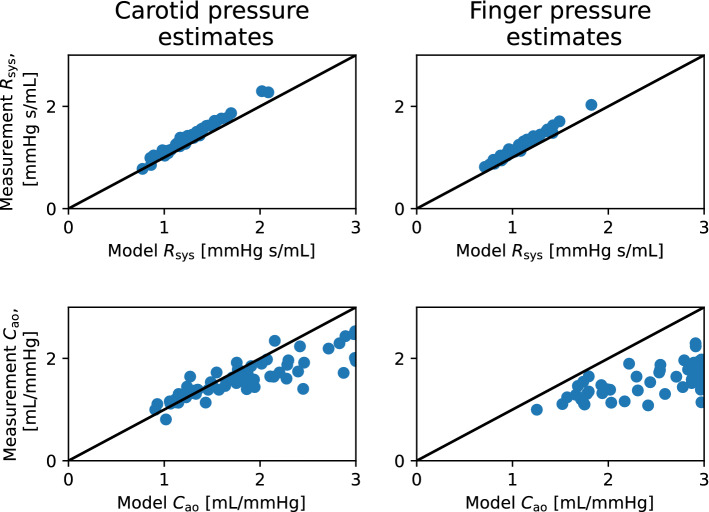
The scatter plots of open-loop model parameter estimates for total peripheral resistance $R_{\text{sys}}$ (top panels) and arterial compliance $C_{\text{ao}}$ (bottom panels), compared to estimates from conventional methods, using the data sets with the carotid (left panels) and the finger pressure (right panels) waveforms. Model estimates are on the x-axes, while conventional estimates are on the y-axes

To assess the credibility of arterial parameter estimates, we compared model estimates to more conventional estimation techniques for $C_{\text{ao}}$ and $R_{\text{sys}}$ as expressed by equations (8) and (9), respectively. Figs. 7–8 show the results for the parameter comparisons in scatter plots for the closed- and open-loop models, respectively. The Pearson correlation coefficient (*r*) was calculated for all comparisons as shown in Table 3. The correlation coefficient and confidence intervals were found by the corr() function in the Pingouin Python library. Estimates for $R_{\text{sys}}$ had a high degree of correlation to the other estimation method, while $C_{\text{ao}}$ showed a moderate amount of correlation. While both models yielded similar results, the measurement location for the pressure waveform does affect the results for $C_{\text{ao}}$ reducing the correlation.

**Table 3:** Correlation coefficients for parameters estimated by the local optimization approach compared to estimates from more conventional methods. Hence, the correlations are found between ${C}_{\text{ao}}$ and $\tilde{C}_{\text{ao}}$ for arterial compliance, while $R_{\text{sys}}$ versus $\tilde{R}_{\text{sys}}$ yield the correlation for peripheral resistance. The *p*-value and 95% confidence interval (95% CI) was obtained by a two-tailed t-test

Parameter	Model	Pressure	Correlation	p-value	95% CI
		waveform	coefficient, *r*		
$R_{\text{sys}}$	Closed-loop	Finger	0.990	$p<1.0e-41$	[0.98, 0.99]
$R_{\text{sys}}$	Closed-loop	Carotid	0.994	$p<1.0e-59$	[0.99, 1.00]
$R_{\text{sys}}$	Open-loop	Finger	0.987	$p<1.0e-39$	[0.98, 0.99]
$R_{\text{sys}}$	Open-loop	Carotid	0.988	$p<1.0e-50$	[0.98, 0.99]
$C_{\text{ao}}$	Closed-loop	Finger	0.601	$p<1.0e-5$	[0.39, 0.75]
$C_{\text{ao}}$	Closed-loop	Carotid	0.864	$p<1.0e-18$	[0.78, 0.92]
$C_{\text{ao}}$	Open-loop	Finger	0.647	$p<1.0e-6$	[0.45, 0.78]
$C_{\text{ao}}$	Open-loop	Carotid	0.852	$p<1.0e-17$	[0.76, 0.91]

The scatter plots of these variables can be seen in Figs. 7 and 8

### Quality of optimization results

### Closed-loop model

**Fig. 9:**
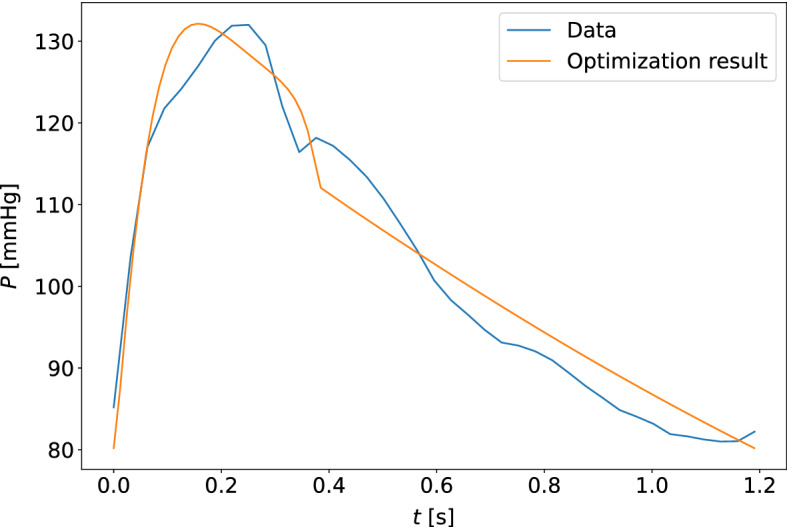
The model-optimized pressure curve based on the mean of the best parameter estimates for the individual is shown with the raw data to which it is optimized for a participant. The model predictions are based on the final averaged parameter estimate for each participant. This is an example of a typical waveform fit for the closed-loop model and the carotid pressure waveform

**Fig. 10:**
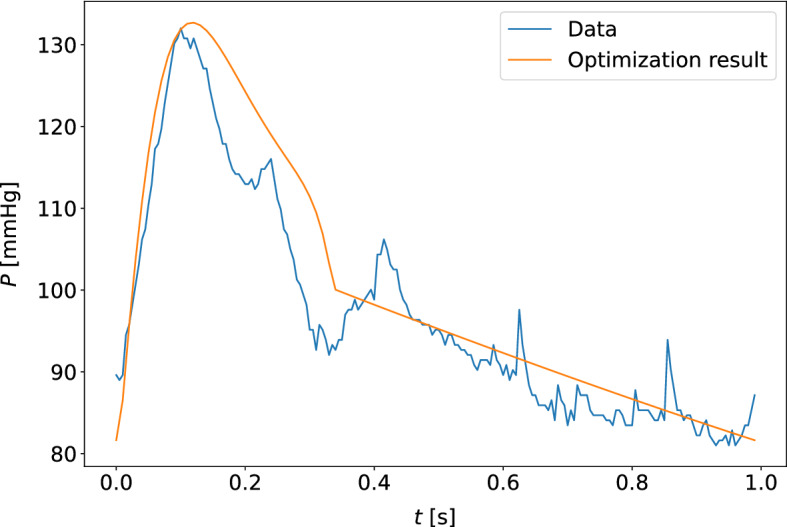
The model-optimized pressure curve based on the mean of the best parameter estimates for the individual is shown with the raw data to which it is optimized for a participant. This is an example of a typical waveform fit for the closed-loop model and the finger pressure waveform

**Fig. 11:**
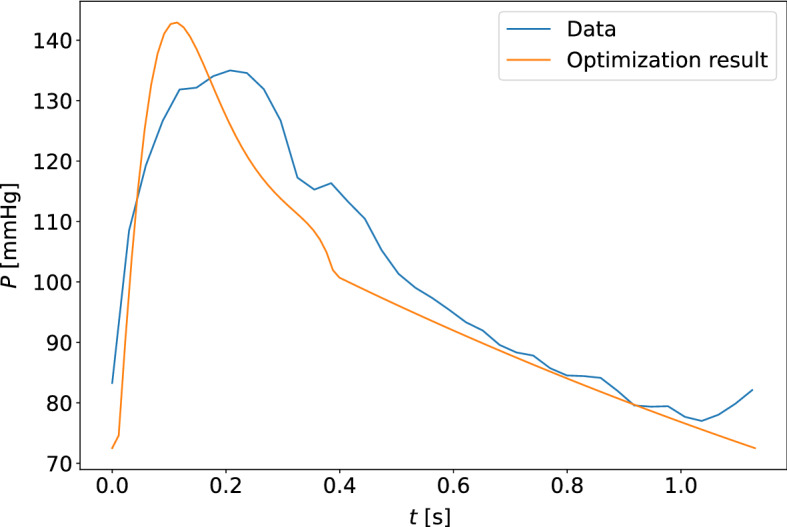
The model-optimized pressure curve based on the mean of the best parameter estimates for the individual is shown with the raw data to which it is optimized for a participant. The sample shown is for the measurement sample with the highest cost function value when compared to other participants and measurement days. This is a waveform adapted to the closed-loop model with the carotid pressure waveform

**Fig. 12:**
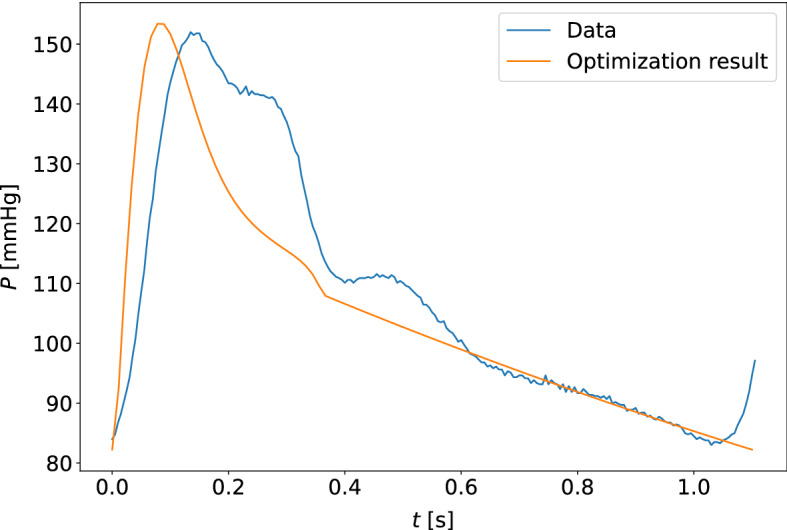
The model-optimized pressure curve based on the mean of the best parameter estimates for the individual is shown with the raw data to which it is optimized for a participant. The sample shown is for the measurement sample with the highest cost function value when compared to other participants and measurement days. This is a waveform adapted to the closed-loop model with the finger pressure waveform

The quality of model optimization was assessed by the unweighted percentage errors of the measurements in the cost function (equation (5)). The results are shown in Table 4, for estimates based on the carotid and finger pressure waveforms for all measurement days separately and collectively. The upper and lower quartiles for the percentage errors along the data points of the pressure and flow waveforms were computed, and the median values for these quartiles across all participants at different choices of measurement days are given in the table. Typical examples of the quality of waveform fits can be found in Figs. 9 and 10. The worst optimized samples according to cost function value can be found in Figs. 11 and 12.

**Fig. 13:**
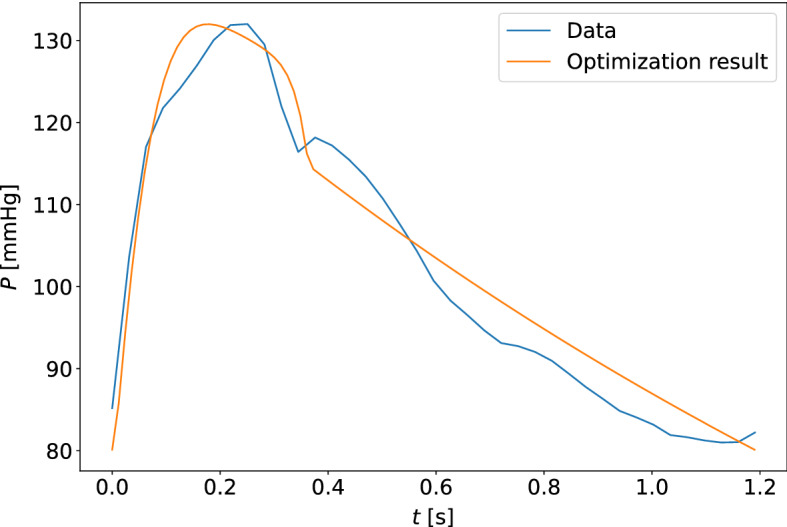
The model-optimized pressure curve based on the mean of the best parameter estimates for the individual is shown with the raw data to which it is optimized for a participant. This is an example of a typical waveform fit for the open-loop model and the carotid pressure waveform

**Fig. 14:**
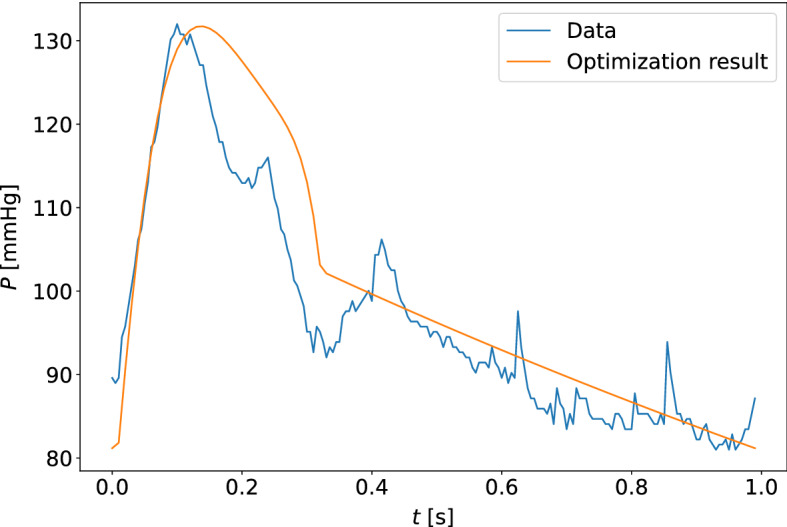
The model-optimized pressure curve based on the mean of the best parameter estimates for the individual is shown with the raw data to which it is optimized for a participant. This is an example of a typical waveform fit for the open-loop model and the finger pressure waveform

**Fig. 15:**
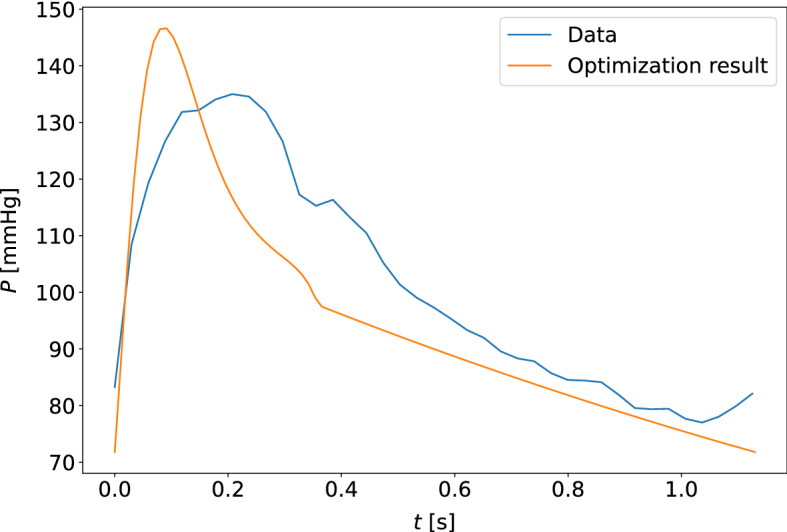
The model-optimized pressure curve based on the mean of the best parameter estimates for the individual is shown with the raw data to which it is optimized for a participant. The sample shown is for the measurement sample with the highest cost function value when compared to other participants and measurement days. This is a waveform adapted to the open-loop model with the carotid pressure waveform

**Fig. 16:**
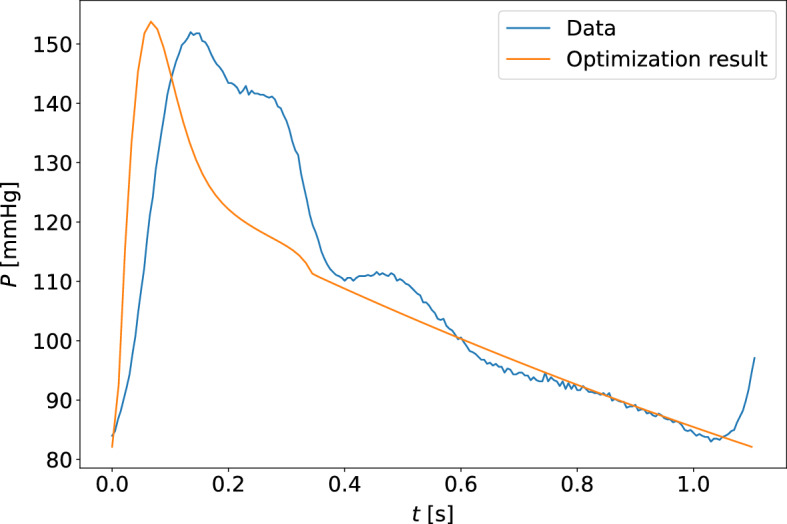
The model-optimized pressure curve based on the mean of the best parameter estimates for the individual is shown with the raw data to which it is optimized for a participant. The sample shown is for the measurement sample with the highest cost function value when compared to other participants and measurement days. This is a waveform adapted to the open-loop model with the finger pressure waveform

**Table 4:** Percentage errors between measurements and model outputs optimized using the closed-loop model with carotid pressure waveforms. These residual statistics are computed across all individuals, i.e. they are not grouped by individual, but by measurement day. The mean and standard deviations of the absolute residuals are given. *P*_sys_ is the systolic aortic pressure, *P*_dia_ is the diastolic aortic pressure, and SV is the stroke volume, and MVP is the mean venous pressure. pWF_x_, and qWF_x_ indicates the median value across all measurements for the x^th^ percentile percentage error of the waveform residuals for a single measurement (pWF_x_ for pressure and qWF_x_ for flow)

Pressure	Meas. day	$P_{\text{sys}}$	$P_{\text{dia}}$	$\text{SV}$	$\text{MVP}$	pWF$_{75\%}$	pWF$_{25\%}$	qWF$_{75\%}$
		[%]	[%]	[%]	[%]	[%]	[%]	[%]
Carotid	All	$1.62 \pm 1.71$	$1.38 \pm 1.36$	$2.32 \pm 2.38$	$6.71 \pm 9.13$	5.03	1.65	26.63
Carotid	1	$1.82 \pm 1.6$	$1.4 \pm 1.34$	$2.4 \pm 2.06$	$6.04 \pm 7.74$	4.94	1.71	22.69
Carotid	2	$1.95 \pm 2.09$	$1.63 \pm 1.69$	$2.92 \pm 2.81$	$8.7 \pm 9.96$	5.32	1.61	30.68
Carotid	3	$0.84 \pm 0.9$	$1.0 \pm 0.69$	$1.34 \pm 1.88$	$4.8 \pm 9.76$	4.52	1.56	25.52
Finger	All	$0.61 \pm 0.79$	$0.72 \pm 0.91$	$1.29 \pm 1.54$	$3.4 \pm 4.21$	5.52	1.48	25.28
Finger	1	$0.61 \pm 0.72$	$0.74 \pm 0.95$	$1.39 \pm 1.63$	$4.15 \pm 5.22$	5.3	1.46	18.7
Finger	2	$0.72 \pm 1.01$	$0.83 \pm 1.1$	$1.67 \pm 1.82$	$3.74 \pm 3.96$	5.65	1.4	30.3
Finger	3	$0.47 \pm 0.54$	$0.56 \pm 0.6$	$0.72 \pm 0.85$	$2.15 \pm 3.03$	5.74	1.79	22.64

### Open-loop model

The quality of model optimization were assessed by the unweighted residuals of the clinical indices in the cost function (equation [5)] in Table 5, for estimates based on the carotid and finger pressure waveforms for all measurement days separately and collectively. Waveform percentage error quartiles and their median value across all participants are given in the table, equivalently as for the closed-loop model. Typical examples of the quality of waveform fits can be found in Figs. 13 and 14. The worst optimized samples according to cost function value can be found in Figs. 15 and 16.

**Table 5:** Percentage errors between measurements and model outputs optimized using the open-loop model with carotid pressure waveforms. These residual statistics are computed across all individuals, i.e. they are not grouped by individual, but by measurement day. The mean and standard deviations of the absolute residuals are given. *P*_sys_ is the systolic aortic pressure, *P*_dia_ is the diastolic aortic pressure, and SV is the stroke volume. pWF_x_, and qWF_x_ indicates the median value across all measurements for the x^th^ percentile percentage error of the waveform residuals for a single measurement (pWF_x_ for pressure and qWF_x_ for flow)

Measurement	Meas. day	$P_{\text{sys}}$	$P_{\text{dia}}$	$\text{SV}$	pWF$_{75\%}$	pWF$_{25\%}$	qWF$_{75\%}$
	[%]	[%]	[%]	[%]	[%]	[%]	[%]
Carotid	All	$0.99 \pm 1.3$	$1.07 \pm 0.87$	$2.82 \pm 4.27$	4.64	1.38	31.24
Carotid	1	$1.03 \pm 1.13$	$1.04 \pm 0.6$	$2.47 \pm 3.54$	4.27	1.42	30.62
Carotid	2	$1.13 \pm 1.62$	$1.25 \pm 1.16$	$3.37 \pm 4.75$	5.14	1.15	34.05
Carotid	3	$0.71 \pm 1.04$	$0.86 \pm 0.69$	$2.51 \pm 4.67$	4.66	1.72	29.65
Finger	All	$0.56 \pm 1.12$	$0.65 \pm 1.33$	$1.44 \pm 2.29$	5.73	1.57	27.0
Finger	1	$0.79 \pm 1.3$	$0.88 \pm 1.54$	$1.95 \pm 2.55$	5.86	1.68	31.9
Finger	2	$0.65 \pm 1.35$	$0.68 \pm 1.62$	$1.73 \pm 2.74$	5.42	1.47	30.99
Finger	3	$0.21 \pm 0.13$	$0.35 \pm 0.38$	$0.52 \pm 0.67$	5.46	1.78	23.68

## Discussion

The variation in parameter estimates for individuals were consistently smaller than the variability in the same parameters for the whole study population, as shown in Figs. 2 and 3. Consequently, this study shows that it is possible to estimate the model parameters for individuals and separate them from parameters of other individuals in the population for the presented estimation heuristic.

The estimates for $C_{\text{sv}}$, and $V_{\text{tot}}$ were subject to the most variability in the population regardless of using the pressure waveform from the carotid or finger artery for the closed-loop model. However, for the open-loop model $E_{\text{max}}$, $C_{\text{ao}}$, and $Z_{\text{ao}}$ were the most variable parameters, but the variability was similar in all three parameters. This study indicates that the estimates using the carotid pressure waveform are more or equally stable for the individual than using the finger pressure waveform, as seen in Figs. 2 and 3. This same pattern is not as apparent in the open-loop model, which may be due to either the parameter space having fewer dimensions or the altered model structure itself. Figures 4 and 5 show that the estimates for the individual had low variation, which was far smaller compared to the population IQR. This suggests that high variability in some model parameters, such as venous compliance, does not affect model outputs substantially. IQRs calculated for the closed-loop model using carotid pressure exhibits very little variability for the individual, indicating that in a majority of cases there is a well-defined local or global minimum which the optimization algorithm chooses. For the same results using finger pressure, there is a higher level of variability, which indicates that a single minimum is harder to obtain in this case.

Examination of results presented in Figs. 2 and 3 shows that the majority of multiple day IQRs were smaller than the population IQR. The only case this was not true was for $t_{peak}$ in the open-loop model using the finger pressure waveform. This parameter is heart rate dependent which changes from beat-to-beat, hence it is not surprising that this parameter could experience variation over all measurement days similar to the population IQR. The $t_{\text{peak}}$ is related to the heart rate of the signal as the length of systole varies with heart rate. Although measurements were performed in a state of rest, it is not guaranteed that everyone had reached their true resting heart rate on each measurement day. For the other parameters, we saw that due to the variability in the multiple-day IQR the standard deviations show that this variability will grow beyond the value of the population IQR for some participants. This means that we potentially could calculate rather large changes from one measurement day to the next. Whether this is realistic remains to be examined, but it can be hypothesized that physical activity induced changes in model parameters larger than the population IQR are unrealistic during a 12-week clinical trial. This would be unrealistic for most of the parameters, aside from $t_{\text{peak}}$. However, the variation in single-day IQR is far lower compared to the multiple-day IQR, in most cases. Exceptions can be noted for $C_{\text{ao}}$, $V_{\text{tot}}$, and $Z_{\text{ao}}$ based on finger pressure where the first and third quartiles overlap for the multi-day and the single-day IQR. This indicates that the single and multi-day IQR may be equally high in some participants, and that changes in these parameters may not in many cases be trusted to be changes caused by the data, and may be artifacts of the numerical uncertainties of the estimation method. This would especially be a challenge for detecting small changes in the individual compared to the variation over the population.

The individual estimates were computed as the mean of the estimates with a cost function value lower than the mean of the final 20 parameter estimate sets, see Sect. Parameter estimation. This allowed the amount of samples used in the computation of the mean parameter estimates to vary. Furthermore, there is a possibility that choosing only the best fitted parameter estimate provides better results in some cases. The averaging over different solutions was chosen to account for sensitivity of the exact location of the cost function minimum due to noise and model structure insufficiency. If, for example, noise causes multiple smaller minima, or a displacement of the local minimum compared to the unperturbed cost function minimum, having multiple estimates in this region and averaging them may mitigate these noise effects. However, using the averaging procedure without bounds for the problem sometimes introduced much higher variability in the resulting solutions, and the averaging would yield parameter values which would not recreate the data well. In contrast, with bounds, we found that the averaged parameters provide acceptable solutions as seen in the table and figures of Sect. Quality of optimization results. The estimates for systolic and diastolic pressures are more accurate for the finger pressure-based estimates since they were more heavily weighted due to more data points since the finger pressure was sampled at a higher frequency than the carotid pressure. We cannot be certain that the algorithm has found the global minimum using this procedure, but it is also a possibility that the global minimum does not correspond the most physiologically accurate combination of parameters. The averaging of solutions around a local minimum gives some information about solutions that are almost as good as the local minimum itself and may be just as physiologically viable. Should the local minimum not be the global minimum, then it is still a minimum which provides a good description of the data as observed in Tables 4 and 5.

In Fig. 6, it is demonstrated that the mean estimates from for the two models only showed small differences based on choice of pressure waveform. A notable exception was found for $C_{\text{ao}}$, for which the finger pressure-based estimates were statistically significantly higher in both model formulations. This may be explained by the finger pressure waveform having a flatter slope during diastole. The $t_{peak}$ was another notable exception, which was estimated with a small difference between waveforms. As previously noted, the corresponding heart rates of the two waveforms could be different. This could potentially contribute to some variation in estimates. The paired sample t-tests between other combinations of model formulations and pressure waveforms are shown in the supplementary materials. The finger pressure was not transformed into a more central pressure by a transfer function, as it was seen as interesting to compare the distal measurement to the carotid waveform which is a more proximal measurement. A question for future work is to answer whether the change in the compliance estimated using the two pressure signals, will change similarly given the same stimulus.

There are no other very large differences in mean parameter estimates between the two models. Therefore, the venous compartment in the closed-loop formulation does not seem to affect the other model estimates to a large extent. Also, it seems like the effect of adjusting the total stressed blood volume can be counteracted by appropriately tuning the venous compliance. Hence, there seems to be little gain of adding these compartments from a parameter estimation perspective.

Two models were applied in this study. Although the closed-loop model may give more insight into the physiology, ventricular filling and fluid distributions of different states and individuals, some parameters could have interacted and caused difficulty in reaching the correct minimum for the cost function in the parameter space. For example, multiple parameters which were all influential on the ventricular filling properties in the model could have caused optimization challenges when adapting all of them to tune the filling properties according to the optimization data set which contained both noise and model discrepancy. One or both of the models may be practically unidentifiable for the data used in this analysis, which could result from such parameter interactions. Specifically, the high variability of stressed volume and venous compliance in the closed-loop model may be the result of such a situation as a higher blood volume would increase the pressure of the closed-loop system, while an increased compliance would accommodate the increased volume and counteract the pressure increase. Similarly, previous work found that the aortic impedance parameter was among the least influential parameters of the aortic pressure waveform [22]. Insensitivity can lead to practical unidentifiability and thus variability in estimates of $Z_{\text{ao}}$ may only reflect this and not any meaningful changes in the hemodynamic state. For the remaining parameters, we have focused on comparing the variability in individual estimates to those of the whole group (Figs. 2, 3) and further compared estimated values to clinical indices to further investigate the consistency of the estimates (Figs. 7, 8). A more formal practical identifiability analysis would be beneficial, but would require a good characterization of the expected variability in measurements and model discrepancy, and this would require same-day repeated measurements of individuals to characterize measurement variation. Further, a required level of precision must be specified for each parameter value. The multiple day IQR found in this study can be interpreted as a conservative estimate of the precision achievable as the repeated measurements included are expected to correspond to changed parameter values in many cases as the measurements are over 12 weeks while increasing physical activity.

The estimated parameter combinations were not assessed in terms of how credible they are to be found in real individuals aside from being bounded by values taken from the literature. By estimating $R_{\text{sys}}$ as $\mathrm {MAP/CO}$ and $C_{\text{ao}}$ as $\mathrm {SV/PP}$, we found high positive correlation between these estimates and the model-predicted resistance. However, this was not the case for $C_{\text{ao}}$, as seen in Figs. 7, 8 where the correlation was lower but still moderately high. The Pearson correlation coefficients are shown in Table 3, and indicate high correlation for $R_{sys}$ in all cases, even if the conventional method estimates are not used for sampling initial parameter guesses for the model optimization.

We focused on using single heart beat cycles of data. The data comprised either synchronized waveforms as for the finger pressure waveform and LVOT flow, or as a single averaged waveform as for pairing the carotid waveform with the LVOT flow data. Colebank et al. and Marquis et al. used an approach where optimization is performed for multiple consecutive cycles at the same anatomical locations which is a convincing approach for accounting for beat-to-beat variations [6, 7], but this requires a large amount of continuous waveform data. We did not do any analysis on the impact of using more than one cycle of data, but previous examinations show that one cycle should be sufficient under ideal conditions for synthetic data [22].

By scaling the waveforms by ABPM systolic and diastolic values, some of the daily variability of measurements should be accounted for. While the waveform shape itself was subject to noise, it may also have been subjected to other perturbations and daily variability through changes in, for example, respiration patterns and heart rate. These effects should be partially accounted for in the averaged carotid waveform. The finger pressure waveforms did not benefit from the same effect as they were not averaged, but were on the other hand synchronized to the simultaneously recorded flow cycles.

Even though the model captures the approximated aortic pressure values reasonably well, this is a highly simplified model of the cardiovascular system with some limitations. Firstly, the model is geometryless, which means that it ignores all personalizable traits relying on spatial geometry more specific than a global compartment of vessels. Despite this, we still observe that the model is able to capture total peripheral resistance, extreme pressures and stroke volumes relatively well, at least when compared to conventional estimation methods for the parameters and the raw data used in the cost function. Secondly, the model neglects the inertance of the vessels and combined with its 0-dimensional nature it therefore ignores potential reversal of blood flow and reflected wave propagation. This makes the model unable to fully describe some features of central pressure waveforms, such as the dicrotic and anacrotic notch. Thirdly, other relevant physiological functions which affect and regulate the cardiovascular system, such as neural, respiratory, renal, metabolism, and gas exchange have been neglected. Any diseases which are not detectable by changes in the systemic hemodynamics and the left ventricle will not be detectable by this model. The parameter ranges that bound the optimization should allow for some types of heart disease and both normal and hypertensive ranges of blood pressure. Any disease or condition which is described by a combination of parameters which needs to exceed one or more of these ranges may not be possible to detect by this personalization algorithm. For the case of monitoring hypertension and possibly related heart remodeling and disease such as heart failure, the model may be able to capture this combined remodeling. However, this has not been investigated by the authors and is beyond the scope of this paper. The ejection fraction (EF) of the model is not necessarily realistic as the ventricular volume intercept is set to a volume of 0. Consequently, if heart failure is to be detected it would likely be more reliably detected by other measures than EF, such as cardiac output or contractility. Lastly, as the inertance is neglected and valve resistances are not explicitly personalized, this model will not account for cardiac valve diseases or leakage.

Some of the synchronized finger pressure waveforms were subject to a high level of noise, while still retaining some characteristic waveform features. This was a drawback for the optimization as it made it more difficult to detect changes and probably contributed to increased variability in estimates. Despite this, we observed similar mean estimates for most model parameters using both finger and carotid waveforms. The only major difference was found in $C_{ao}$ which can be partially be explained by the flatter diastolic slope of the finger pressure waveforms. The finger pressure waveform does experience pressure amplification and distortion of the waveform compared to more central pressures such as the carotid pressure waveform. Therefore, we may have expected to see some differences in the mean parameters as well. In Eq. 5, the clinical indices get additional weighting to increase their priority in the optimization scheme. If the weights were removed the optimization procedure would be able to recreate the waveforms even closer, but also allow larger discrepancies in the remaining terms of the cost function. This could in turn have caused even larger discrepancies between results from the different choices of pressure waveform, but for this investigation we chose a balance between adaption to waveform or more clinically relevant measures such as systolic and diastolic pressures.

## Conclusion

Model personalization was performed for blood pressure and echocardiography data collected from 25 participants in an physical activity motivational study were used for model optimization.

Mean parameter estimates were practically equivalent across both model formulations and for both choices of waveforms, except for a few cases. The $C_{\text{ao}}$ parameter was found to have a higher value on average when estimated using the finger pressure waveform as compared to the carotid waveform, regardless of model choice. For both models, the estimates for arterial resistance and compliance were found to correlate at least moderately well ($r>$ 0.60) with other conventional estimation methods (Additional files 1, 2).

Changes in pressure and flow waveforms, as well as SV are reproduced reasonably well by both models for the estimated parameters. Using the closed-loop model did not prove to aid the ability to resolve single participants’ parameter estimates from the model population compared to the open-loop model. This supports using the open-loop model formulation for further efforts in personalizing the ventricle and arterial compartments of a lumped parameter model.

Resolving parameter changes for individuals and distinguishing these changes from the population seems feasible given the IQR values, assuming the real changes are sufficiently large to not be lost in the personal estimation variability. Questions for further research are whether or not these changes are realistic or a product of noise, insufficient data, or uncertainty introduced in the estimation procedure. Whether the data are sufficient to detect cardiovascular remodeling given the recorded exercise stimulus remains to be investigated.

### Supplementary Information

**Additional file 1. Table S1.** Two-tailed t-tests for parameter means using different models and pressure waveforms. Parameter values averaged over different samples of measurements for both model formulations and choices of data sets. Standard deviations are presented in parentheses. The p-value and 95% confidence interval (CI95%) are obtained by a two-tailed t-test for paired data comparing the mean parameter values using the finger and carotid pressure for the same model formulation, or using the same pressure waveform but different model formulations. “Group” indicates which combination of model and pressure waveform is used to generate the data for the group. “CL” indicates the closed-loop model, and “OL” signifies the open-loop model. “-C” indicates the carotid pressure waveform, and “-F” indicates the finger pressure waveform. The parameters included in the analysis are systemic arterial compliance (Cao), total peripheral resistance (Rsys), time of peak elastance in the left ventricle (tpeak), characteristic aortic impedance (Zao), and maximal left-ventricular elastance (Emax).

**Additional file 2: Table S1.** Two-tailed t-tests for parameter means using different models and pressure waveforms. Parameter values averaged over different samples of measurements for both model formulations and choices of data sets. Standard deviations are presented in parentheses. The p-value and 95% confidence interval (CI95%) are obtained by a two-tailed t-test for paired data comparing the mean parameter values using the finger and carotid pressure for the same model formulation, or using the same pressure waveform but different model formulations. “Group” indicates which combination of model and pressure waveform is used to generate the data for the group. “CL” indicates the closed-loop model, and “OL” signifies the open-loop model. “-C” indicates the carotid pressure waveform, and “-F” indicates the finger pressure waveform. The parameters included in the analysis are systemic arterial compliance (Cao), total peripheral resistance (Rsys), time of peak elastance in the left ventricle (tpeak), characteristic aortic impedance (Zao), and maximal left-ventricular elastance (Emax).

## Data Availability

The data sets and code used and/or analyzed during the current study can be provided by the corresponding author upon reasonable request.

## Appendix

### Model equations

#### Closed-loop model

This subsection is largely a repetition of details described in [22]. The closed-loop model comprised a system of non-linear ordinary differential equations (ODEs) describing the volume state variable of the three model compartments. The dynamic elastance function of the heart contributes a source term to the system of ODEs:

10 $$\begin{aligned} \begin{aligned} \frac{dV_{sa}}{dt}&= C_{sa} \frac{dP_{sa}}{dt} = Q_{lvao} - Q_{sys} \\ \frac{dV_{sv}}{dt}&= C_{sv} \frac{dP_{sv}}{dt} = Q_{sys} - Q_{svlv} \\ \frac{dV_{lv}}{dt}&= Q_{svlv} - Q_{lvao}. \\ \end{aligned} \end{aligned}$$

The two remaining state variables, pressure and flow, are mainly modeled by linear relationships as expressed below:

11 $$\begin{aligned} \begin{aligned} V_{sa}&= C_{sa} P_{sa} \\ V_{sv}&= C_{sv} P_{sv} \\ P_{lv}&= E(t) V_{lv} + P_{th}(t) \\ E(t)&= (E_{\max } - E_{\min }) e(t) + E_{\min } \\ P_{ao}&= \max {[P_{sa}, P_{lv}]} \\ Q_{lvao}&= I(P_{lv}> P_{sa}) \frac{P_{lv} - P_{sa}}{Z_{ao}} \\ Q_{svlv}&= I(P_{sv} > P_{lv}) \frac{P_{sv} - P_{lv}}{R_{mv}} \\ Q_{sys}&= \frac{P_{ao} - P_{sv}}{R_{sys}}. \end{aligned} \end{aligned}$$

The indicator function *I*(*x*) has the value 1 when the argument *x* is true and 0 when *x* is false. The activation function $e(\tau )$ is defined as:

12 $$\begin{aligned} e(\tau ) = \alpha \times \frac{(\tau /a_1)^{n_1}}{1 + (\tau /a_1)^{n_1}} \times \frac{1}{1 + (\tau /a_2)^{n_2}}, \end{aligned}$$

where $\tau$ is position in the cardiac cycle between the end of the last diastolic period and the end of the next diastolic period $\tau =1$. The parameters $a_1$ and $n_1$ determine the shape of contraction and $a_2$ and $n_2$ determine the shape of relaxation of the elastance curve and the timing of peak elastance. The choice of values for these parameters are identical to those of Stergiopulos et al [24]. We wrote the parameter values for $a_1$ and $a_2$ in terms of the ratio of $\frac{t_{peak}}{T}$, and set $\alpha = 1.672$, to ensure normalization of the curve. $t_{peak}$ describes the time of peak ventricular elastance, and therefore determines when the left-ventricular elastance *E*(*t*) reaches $E_{max}$. The intrathoracic pressure function $P_{th}$ describes the external pressure effects on the ventricular muscle aside from pressure gradients inside the blood vessels and is here modeled as a constant of $P_{th}(t) = -4$ mmHg. Otherwise, the parameters are defined as in Table 1.

The $V_{tot}$ parameter describes total stressed volume and is enforced by setting initial compartment volumes and pressures such that the total stressed volume equals the parameter value. The model was demonstrated to conserve the volume and hence the total blood volume will not change. The initial volumes are set according to the equations:

13 $$\begin{aligned} {\begin{matrix} &{} V_{ao,0} = C_{ao} \ P_{ao,0}, \ \text{and} \\ &{} P_{sv,0} = \frac{V_{tot} - V_{ao,0} - V_{lv,0}}{C_{sv}}, \end{matrix}} \end{aligned}$$

where the initial aortic pressure is set to $P_{ao,0} = 100$ mmHg, and initial left-ventricular volume is set to $V_{lv,0} = 100$ mL. The initial venous pressure is denoted by $P_{sv,0}$.

#### Open-loop model

The open-loop model describes two compartments, the left ventricle and arteries. The heart compartment is identical to the one described for the closed-loop model. The state equations describing the model are as follows:

14 $$\begin{aligned} \begin{aligned} \frac{dV_{sa}}{dt}&= C_{sa} \frac{dP_{sa}}{dt} = Q_{lvao} - Q_{sys} \\ \frac{dV_{lv}}{dt}&= Q_{svlv} - Q_{lvao}. \\ \end{aligned} \end{aligned}$$

The state variables pressure, flow and volume are mainly modeled by linear relationships as seen here:

15 $$\begin{aligned} \begin{aligned} V_{sa}&= C_{sa} P_{sa} \\ P_{lv}&= E(t) V_{lv} + P_{th}(t) \\ E(t)&= (E_{\max } - E_{\min }) e(t) + E_{\min } \\ P_{ao}&= \max {[P_{sa}, P_{lv}]} \\ Q_{lvao}&= I(P_{lv}> P_{sa}) \frac{P_{lv} - P_{sa}}{Z_{ao}} \\ Q_{svlv}&= I(P_{sv} > P_{lv}) \frac{P_{sv} - P_{lv}}{R_{mv}} \\ Q_{sys}&= \frac{P_{ao} - P_{sv}}{R_{sys}}, \end{aligned} \end{aligned}$$

where *e*(*t*) is as defined in Equation (12), while $P_{\text{sv}} = 6.0$ mmHg is now a fixed quantity. Now the initial volumes are set as

16 $$\begin{aligned} {\begin{matrix} &{} V_{ao,0} = C_{ao} \ P_{ao,0}, \ \text{and} \\ &{} V_{lv,0} = 100 \ \text{mL}, \end{matrix}} \end{aligned}$$

where the initial aortic pressure is set to $P_{ao,0} = 100$ mmHg.

### Optimization algorithm details

The Trust Region Reflective Algorithm (TRRA) as implemented in SciPy version 1.7.1 was applied for the parameter optimization procedure [29]. In particular, the implementation of the algorithm as expressed through the function scipy.optimize.least_squares() was used. We executed this function given the arguments below and a list of initial parameter guesses as sampled from Eq. (3). The function specific parameters passed to the TRRA to set the accuracy of the method were set to $xtol = 2.3 \cdot 10^{-16}, ftol = 2.3 \cdot 10^{-16}, gtol = 2.3 \cdot 10^{-16}$ and $diff\_step = 1.0 \cdot 10^{-3}$.

The uniform distributions from which the first 30 initial parameter guesses are sampled were bounded by the upper and lower bounds presented in 6. For the next 20 initial parameter guesses the sampling was made from the uniform distribution with bounds expressed as (4). In Eq. (4), $\theta _{min,i}$ refers to the initial parameter guess form the first 30 guesses which optimized the solution with the minimal cost function value from that first set of guesses. The same bounds were upper and lower limits for parameters in both sampling distributions.

All parameters from Table 1 were sampled, except *T* which was always set to the heart beat period of the given waveform, as well as $E_{\text{min}}$ and $R_{\text{mv}}$ which were fixed to predetermined values. Mitral valve resistance should be a small resistance according to Stergiopulos et al. [24], which we fixed it to 0.02 mmHg s/mL. The value of $E_{\text{min}}$ was set according to apparent phenotype based on blood pressure. Segers et al. estimated values for minimal left-ventricular elastances as 0.03 mmHg/mL for normotensive people [12], while we calculated the same parameter to be 0.034 when taken as the weighted average over all hypertensive groups presented in their study. This value caused difficulties in estimating the left-ventricular elastance by constantly estimating it to the lowest bound for all participants. Therefore the value of 0.06 mmHg/mL as used by Stergiopulos was used instead, but separation was made according to if the systolic blood pressure was above or below 140 mmHg as was similar to the case for the estimates by Segers et al. [12]. For systolic blood pressure below 140 mmHg, $E_{\text{min}} = 0.055$ mmHg/mL was opted for, while we used for values above $E_{\text{min}} = 0.06$ mmHg/mL.

The bounds in Table 6 were set based on many different sources in literature. The study from Vardoulis et al. simulated a wide range of total arterial compliances [31], and we used a similar range for our concept of arterial compliance, but it was slightly widened as finger pressure estimates tend to have a low diastolic slope, which might indicate a high compliance. Central venous compliance is often estimated to be 30 times the value of arterial compliance [32, 33], and therefore, we used estimates of the venous compliance 10–30 times the arterial compliance values to make the widest range possible. For $E_{max}$ we used the body surface area (BSA) indexed results from a study by Bombardini et al. which estimated end-systolic elastances for patients in both disease and health to estimate a wide range to use as bounds for sampled elastances [34]. For $R_{\text{sys}}$, Chantler et al. estimated vascular resistance in groups men and women with normo- or hypertension [35]. We considered all four groups, the group with the lowest mean value minus two standard deviations was taken as the lowest bound, and the group with the highest mean was used in the opposite direction to determine the highest bound. $t_{peak}$ was bounded using results from studies by Weissler et al. and Mertens et al., which both measured systolic timing properties of the heart [36, 37]. Their measurements of the $QS_2$ period of the heart ejection time determined by electromechanical considerations were taken as and indication of how large the allowed range for $t_{\text{peak}}$ should be, and hence the bounds were determined by a technique similar to how it was done for $R_{\text{sys}}$. The lower bound was lowered further as to accommodate an even wider range. The total stressed blood volume bounds were estimated by taking the measurements of total blood volume from a paper by Feldschuh et al. and applying a formula as demonstrated by Colunga et al. to estimate the total blood volume [5, 38]. This was done by taking the total blood volume value and estimating the total stressed volume fraction by this formula:

17 $$\begin{aligned} V_{\text{tot}} \approx \text{TBV} \cdot (0.13 \cdot 0.27+0.64 \cdot 0.18+0.035 \cdot 0.5), \end{aligned}$$

where the total stressed blood volume $V_{\text{tot}}$ is computed by assuming what fraction of the total blood volume (TBV) can be found in the left ventricle, systemic arteries, and systemic veins, and what fractions of these volumes are assumed to be stressed. The mean blood volumes, of the study groups examined by Feldschuh et al. [38], with the highest and lowest mean values were adjusted by adding or subtracting 3–4 times the standard deviations to find the widest possible range based on these data. Two standard deviations gave volumes which were larger than what was expected to give reasonable results with our model. Finally, the bounds for $Z_{\text{ao}}$ were based on the values reported by Segers et al. [12], but we allowed a range 33 times wider as this was a small parameter compared to the other arterial parameters. The upper range was set to ensure that it likely would be smaller than the total peripheral resistance. The resulting parameter range also corresponds reasonably well with the range found by Segers et al. when optimizing the parameter to multiple data sets for a three-element Windkessel model [39].

The scaling factors used to balance the different terms in the cost functions in (5) are listed in Table 7. The initialization for the random seed using the numpy.random.seed() function from the NumPy libary [40], was set to be 112233 for sampling initial parameter guesses from the distributions defined by (3) and (4).

**Table 6:** All model parameters that were assigned to be personalizable are listed along with their upper and lower bounds. The bounds determine the uniform distributions from which the initial parameter guesses are sampled

Parameter	Upper bounds	Lower bounds	Units
$C_{ao}$	3.0	0.148	$\mathrm {\frac{mL}{mmHg}}$
$C_{sv}$	90.0	1.48	$\mathrm {\frac{mL}{mmHg}}$
$E_{max}$	$\frac{10.48}{\text{BSA}}$	0.5	$\mathrm {\frac{mmHg}{mL}}$
$R_{sys}$	2.963	$\frac{0.917}{\text{BSA}}$	$\mathrm {\frac{mmHg \ s}{mL}}$
$t_{peak}$	$\min (0.442, T)$	$\min (0.15, 0.9 T)$	s
$V_{tot}$	1503.	150.	mL
$Z_{ao}$	0.2	0.001	$\mathrm {\frac{mmHg \ s}{mL}}$

**Table 7:** The scaling factors *K*, which are used to balance and approximately normalize the terms in the specified cost functions. *MVP *- mean venous pressure, *p* - aortic pressure waveform, *pdia* - diastolic brachial pressure, *psys* - systolic brachial pressure, *q* - aortic flow, and *SV* - stroke volume

Symbol	Value	Unit
$K_{MVP}$	5.0	mmHg
$K_{p}$	100.0	mmHg
$K_{pdia}$	80.0	mmHg
$K_{psys}$	120.0	mmHg
$K_{q}$	500.0	$\mathrm {\frac{mL}{s}}$
$K_{SV}$	100.0	mL
