# Supplementary material for: Monitoring variability in parameter estimates for lumped parameter models of the systemic circulation using longitudinal hemodynamic measurements
Source: Biomed Eng Online. 2023 Apr 13;22:34. doi: 10.1186/s12938-023-01086-y (PMC10099701; doi:10.1186/s12938-023-01086-y)
Supplement: Supplementary file 2 — Additional file 2: Table S1. Two-tailed t-tests for parameter means using different models and pressure waveforms. Parameter values averaged over different samples of measurements for both model formulations and choices of data sets. Standard deviations are presented in parentheses. The p-value and 95% confidence interval (CI95%) are obtained by a two-tailed t-test for paired data comparing the mean parameter values using the finger and carotid pressure for the same model formulation, or using the same pressure waveform but different model formulations. “Group” indicates which combination of model and pressure waveform is used to generate the data for the group. “CL” indicates the closed-loop model, and “OL” signifies the open-loop model. “-C” indicates the carotid pressure waveform, and “-F” indicates the finger pressure waveform. The parameters included in the analysis are systemic arterial compliance (Cao), total peripheral resistance (Rsys), time of peak elastance in the left ventricle (tpeak), characteristic aortic impedance (Zao), and maximal left-ventricular elastance (Emax). [file 12938_2023_1086_MOESM2_ESM.pdf]

## Additional file

### Two-tailed t-tests for parameter means using different models and pressure waveform

Table 1 contains paired t-test results of comparing the distributions of final parameter values estimated for the same participants on the same measurement days using different models and data. This is repeated for all parameters that are common for both the closed-loop and open-loop models.

| Param. | Group 1 | Mean of group 1 | Group 2 | Mean of group 2 | Mean diff. | p-value | 95% CI for diff. of means | Units |
|---|---|---|---|---|---|---|---|---|
| $C_{ao}$ | CL-F | 1.28(0.25) | CL-C | 0.88(0.24) | 0.40(0.24) | p < 1.e-14 | [0.33 0.47] | $\frac{mL}{mmHg\ m^2}$ |
| $R_{sys}$ | CL-F | 0.55(0.14) | CL-C | 0.63(0.15) | -0.08(0.05) | p < 1.e-14 | [-0.09 -0.07] | $\frac{mmHg\ s}{mL\ m^2}$ |
| $t_{peak}$ | CL-F | 0.36(0.04) | CL-C | 0.40(0.03) | -0.03(0.03) | p < 1.e-07 | [-0.04, -0.02] | s |
| $Z_{ao}$ | CL-F | 0.05(0.01) | CL-C | 0.05(0.01) | 0.00(0.01) | p = 0.2513 | [0., 0.] | $\frac{mmHg\ s}{mL\ m^2}$ |
| $E_{max}$ | CL-F | 1.08(0.24) | CL-C | 1.03(0.19) | 0.05(0.19) | p = 0.1045 | [-0.01, 0.1 ] | $\frac{mmHg}{mL\ m^2}$ |
| $C_{ao}$ | CL-C | 0.88(0.24) | OL-C | 0.86(0.25) | 0.02(0.08) | p = 0.1636 | [-0.01, 0.04] | $\frac{mL}{mmHg\ m^2}$ |
| $R_{sys}$ | CL-C | 0.63(0.15) | OL-C | 0.63(0.15) | -0.01(0.01) | p = 0.0007 | [-0.01, 0. ] | $\frac{mmHg\ s}{mL\ m^2}$ |
| $t_{peak}$ | CL-C | 0.40(0.03) | OL-C | 0.39(0.04) | 0.00(0.02) | p = 0.1283 | [0., 0.01] | s |
| $Z_{ao}$ | CL-C | 0.05(0.01) | OL-C | 0.05(0.01) | 0.00(0.00) | p = 0.0183 | [0., 0.] | $\frac{mmHg\ s}{mL\ m^2}$ |
| $E_{max}$ | CL-C | 1.03(0.19) | OL-C | 0.97(0.26) | 0.06(0.21) | p = 0.0500 | [0., 0.12] | $\frac{mmHg}{mL\ m^2}$ |
| $C_{ao}$ | CL-F | 1.28(0.25) | OL-F | 1.22(0.26) | 0.05(0.08) | p < 1.e-04 | [0.03, 0.08] | $\frac{mL}{mmHg\ m^2}$ |
| $R_{sys}$ | CL-F | 0.55(0.14) | OL-F | 0.55(0.14) | -0.00(0.01) | p < 1.e-05 | [-0.01, 0. ] | $\frac{mmHg\ s}{mL\ m^2}$ |
| $t_{peak}$ | CL-F | 0.36(0.04) | OL-F | 0.36(0.04) | 0.01(0.01) | p = 0.0002 | [0., 0.01] | s |
| $Z_{ao}$ | CL-F | 0.05(0.01) | OL-F | 0.05(0.01) | 0.00(0.00) | p = 0.0005 | [0., 0.] | $\frac{mmHg\ s}{mL\ m^2}$ |
| $E_{max}$ | CL-F | 1.08(0.24) | OL-F | 1.00(0.32) | 0.08(0.20) | p = 0.0132 | [0.01, 0.13] | $\frac{mmHg}{mL\ m^2}$ |
| $C_{ao}$ | OL-F | 1.22(0.26) | OL-C | 0.86(0.25) | 0.36(0.23) | p < 1.e-13 | [0.29, 0.43] | $\frac{mL}{mmHg\ m^2}$ |
| $R_{sys}$ | OL-F | 0.55(0.14) | OL-C | 0.63(0.15) | -0.08(0.05) | p < 1.e-15 | [-0.09, -0.07] | $\frac{mmHg\ s}{mL\ m^2}$ |
| $t_{peak}$ | OL-F | 0.36(0.04) | OL-C | 0.39(0.04) | -0.04(0.04) | p < 1.e-08 | [-0.05, -0.03] | s |
| $Z_{ao}$ | OL-F | 0.05(0.01) | OL-C | 0.05(0.01) | 0.00(0.01) | p = 0.1282 | [0., 0.] | $\frac{mmHg\ s}{mL\ m^2}$ |
| $E_{max}$ | OL-F | 1.00(0.32) | OL-C | 0.97(0.26) | 0.03(0.09) | p = 0.0199 | [0.01, 0.06] | $\frac{mmHg}{mL\ m^2}$ |

Table S1: Parameter values averaged over different samples of measurements for both model formulations and choices of data sets. Standard deviations are presented in parentheses. The p-value and 95% confidence interval (CI95%) are obtained by a two-tailed t-test for paired data comparing the mean parameter values using the finger and carotid pressure for the same model formulation, or using the same pressure waveform but different model formulations. "Group" indicates which combination of model and pressure waveform is used to generate the data for the group. "CL" indicates the closed-loop model, and "OL" signifies the open-loop model. "-C" indicates the carotid pressure waveform, and "-F" indicates the finger pressure waveform. The parameters included in the analysis are systemic arterial compliance ($C_{ao}$), total peripheral resistance ($R_{sys}$), time of peak elastance in the left ventricle ($t_{peak}$), characteristic aortic impedance ($Z_{ao}$), and maximal left-ventricular elastance ($E_{max}$)

.
